# On Antimicrobial Polymers: Development, Mechanism of Action, International Testing Procedures, and Applications

**DOI:** 10.3390/polym16060771

**Published:** 2024-03-11

**Authors:** Saleh Alkarri, Hawra Bin Saad, Maria Soliman

**Affiliations:** 1 School of Packaging, Michigan State University, 448 Wilson Road, East Lansing, MI 48824-1223, USA; 2 Imperial College London, Molecular Sciences Research Hub (MSRH), 82 Wood Lane, London W12 0BZ, UK; 3 SABIC, Urmonderbaan 22, P.O. Box 319, 6167 RD Geleen, The Netherlands

**Keywords:** antimicrobial activity, antimicrobial agents, leachable agents, non-leachable agents, mode of action, testing protocols

## Abstract

The development of antimicrobial polymeric materials has evolved into one of the more promising methods for preventing the growth of microbes and mitigating the spread of infectious diseases in several applications including the health and food packaging sectors. The outbreak of global pandemics, and particularly the recent COVID-19 pandemic, further strengthen the importance of developing such solutions. This review paper presents a fundamental understanding of how antimicrobial polymers are developed, describes the possible surface modification approaches to render polymers with antimicrobial properties, highlights the potential mechanism of action against a range of microorganisms (bacterial, viral, and fungal), and details some of the international standard protocols and procedures to evaluate the antimicrobial properties of modified materials (such as plastics and textiles). In addition, this review paper discusses the toxicity of antimicrobial additives when used in healthcare and food packaging applications.

## 1. Introduction

Scientists have estimated that there is a total of ~1400 known species of pathogens affecting humans, including viruses, bacteria, fungi, protozoa, and helminths. These represents less than 1% of the number of microbes on Earth, yet these pathogens contribute to an average of 16 million deaths per year, yet the majority of these deaths are preventable [1]. The unchecked growth of infectious microbes can not only cause premature deaths but also can bring societies and economies to a standstill, as seen in 2019 with the COVID-19 outbreak. Therefore, since the pioneering discoveries of Louis Pasteur and Robert Koch, researchers, and healthcare systems in general, have developed cures for diseases caused by these infectious microbes. Antibiotics are medicines used to treat people and animals with bacterial infections by either killing the bacteria or inhibiting their growth. Antibiotics are effective at killing many strains of harmful bacteria, but some disadvantages must be considered. Firstly, while antibiotics are effective solutions against bacteria, they are not as effective against viruses. Secondly, certain bacteria have become resistant to at least one out of every three antimicrobial drug categories, which often occurs because patients terminate the antibiotic treatments as soon as external symptoms subsidize or because health care providers overprescribe antibiotics [2,3,4,5]. The three categories of antimicrobial drugs are defined by their activity spectrum and classification as narrow-, broad-, or extended-spectrum agents. Antibiotics falling within the narrow spectrum, such as penicillin G, are more effective on Gram-positive bacteria, whereas antibiotics classified as broad-spectrum, such as tetracyclines and chloramphenicol, are effective on both Gram-positive and Gram-negative bacteria. An extended-spectrum antibiotic is one that, because of chemical modification, affects additional types of bacteria, usually those that are classified as Gram-negative [6]. The Gram stain differentiates organisms within the domain bacteria, based on their cell wall structure. Gram-positive cells possess thick peptidoglycan layers, external to the cell membrane, and Gram-negative cells have thin peptidoglycan layers situated between two membrane layers [7]. The Gram-positive bacteria are more sensitive towards antibacterial substances than Gram-negative bacteria because of their cell wall structure which is protected by an outer membrane [8]. 

The development of new types of antibiotics cannot keep pace with the emergence of new drug-resistant bacterial varieties due to the high costs to research and develop new drugs and the time needed to obtain regulatory clearance [9]. Furthermore, the rapid evolution of antibiotic resistance severely limits the usefulness of new antibiotics as well as the economic return for the years of research and millions of dollars of investment [10]. As a result, many people are infected with multidrug-resistant bacteria every year [11], leading to premature deaths.

Infectious diseases can be spread via contaminated surfaces through the transfer of existing microorganisms to mucous membranes in the body [12]. This is a key issue in hospitals with rising numbers of nosocomial infections and associated deaths [13]. Plastics with antimicrobial properties are one of the solutions to reducing the spread of infections, and the COVID-19 pandemic generated even more interest because of the fear of virus transmission through fomites [14]. Most common plastics have no inherent biocidal properties and thus may be surfaces upon which microbes may persist and become a source of infection [15]. 

One class of antimicrobial polymeric systems is focused on antibiotic or drug release. The aim is the controlled and targeted release of the antibiotic or drug, and the polymer serves as an inert matrix for holding the biocide. In contrast to the antibiotic method that counters infectious microbes, researchers have explored antimicrobial polymers’ unique properties in roles other than as media for “controlled drug release”. Antimicrobial polymers contain substance(s) that kill microorganisms or stop their multiplication and growth. The antimicrobial polymer substances can be engineered to (i) prevent the adhesion of microbes and/or (ii) have functional groups that kill microbes or (iii) contain nano additives that destroy microbes.

These engineered methods have particular advantages. Firstly, they provide a preventive solution that can be applied to stop an infection from arising in the first place. Secondly, polymer containing moieties can be combined with multiple antimicrobial mechanisms that cannot be outwitted by pathogens [16]. Secondly, unlike antibiotics, these polymers lower the potential for the microbes to develop resistance. Thirdly, these polymers could be incorporated with additives that are effective against viruses as well as bacteria. Once developed, these antimicrobial polymers can be easily adapted to wider applications. They can be applied either as a coating to protect working surfaces and medical instruments against microbial growth, or they can be melt-compounded and molded into objects, such as doorknobs, keypads, shopping cart handles, etc, that are exposed to manual handling and are accumulation points for microbes. Plastics with antimicrobial properties could replace consumable disinfectants or could be an additional defense in the war against infectious microbes.

**Figure 1:**
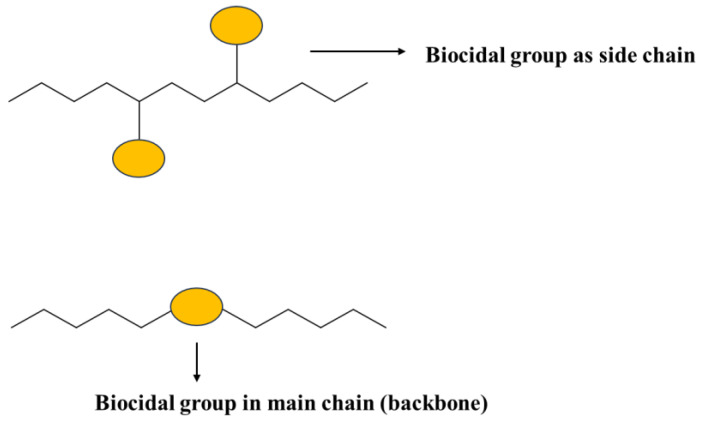
Illustration of biocidal group incorporated in the polymer backbone or as a sidechain.

In contrast to biocide-releasing polymers, “polymeric biocides” are macromolecules with microbe killing “functional groups” in the side chain or in the main-chain backbone (Figure 1). That is, the polymer itself is the microbe killer. Such antimicrobial polymers are designed to imitate the antimicrobial defensive structures created by the immune systems of many organisms to eliminate microorganisms.

Other “biocidal polymers” inhibit the adhesion of microbes or have additives (not antibiotics) that kill microbes. In these cases, the polymer itself may be inert; these biocidal polymers include nanocomposites and blends of an inert polymer + polymeric biocide.

## 2. Polymers

A natural or synthetic material made of macromolecules is a polymer. Examples of natural polymers are wool and silk, while synthetic polymers include vulcanized rubber, polystyrene, and polyethylene [17]. Polymers have unique chemical and physical properties, making them useful in everyday life. For example, vulcanized rubber is used in car tires because of its improved quality and resistance to deformation. This rubber is synthesized using polymerization, whereby constituent monomers react together to form polymer chains. The two general types of polymerizations include either condensation or addition reactions. Condensation polymerization involves multiple condensation reactions between monomers including two or three functions that release small molecules such as water as a side product [18]. Advantages include strength, durability, low density, and chemical stability, while disadvantages include its carbon footprint (Division of Polymeric Materials American Chemical Society, 2002). Examples are silicones and nylon [19]. Addition polymerization involves the recurrent addition of monomers with two or three bonds to form a polymer chains [20]. Advantages include high melting points and rigidity, while drawbacks are depletion of oil and pollution [20]. Examples include polyvinylchloride (PVC) and Teflon [21].

### 2.1. Biocidal-Releasing Polymers

The “biocidal releasing polymers” are either antibiotic or drug-loaded polymers which are mainly used when the patient is already infected. The aim is “controlled drug release” and “targeted delivery” to the infected site. There are five categories of biocide-releasing polymers used for drug release: (i) nanoparticles (NPs) of special polymers, (ii) polymer micelles, (iii) polymer vesicles, (iv) dendrimers, and (v) polymeric hydrogels [22].

### 2.2. Nanoparticles (NPs) of Special Polymers

Nanoparticles (NPs) are unique substances manufactured on a nanoscale, where a minimum of 50% of the particles within the size distribution have one or more external dimensions ranging from 1 nm to 100 nm. Polymeric NPs that are inherently antimicrobial have been used for targeted drug release against cancer cells. These polymeric NPs contrast with what is later described as antimicrobial polymer nanocomposites, which consist of polymers with metal oxide or metal NPs [23,24]. The polymeric NPs are made of self-assembled (hierarchical) structures, and the targeted delivery to infected sites is achieved by controlling the surface chemistry attributes of the resultant formations. The delivery of the drug from the biocide-releasing polymer NPs can occur over days and even weeks. There are some advantages of using biocide releasing polymeric NPs: (i) their ability to protect the drugs from enzymatic breakdown, thus decreasing the amount of drug needed, and reducing its toxicity to the recipient and (ii) their potential ability to overcome bacterial resistance mechanisms [25]. In fact, some polymeric self-assembled NPs use a multimodal mechanism of action to destroy microbes such as (i) outer bacterial membrane destabilization, (ii) inner membrane disturbance, and (iii) disruption of ionic flows across microbe cell walls [26,27].

### 2.3. Polymer Micelles

The “polymer micelles” from the core–shell polymeric NPs are created via the self-organization of block copolymers with both hydrophilic and hydrophobic parts (a combination of hydrophobic and hydrophilic components). The core houses hydrophobic drugs, while the shell is designed to form water-soluble micelles for delivering poor-solubility drugs. In some cases, these polymer micelles have the ability to function as antimicrobial polymers independently, without the need for additional biocidal agents [28]. Biodegradable antimicrobial polymeric micelles made from self-assembled amphiphilic polycarbonate (PC) showed disruption of the bacterial cell wall and membrane resulting in bacterial breakdown [29]. While effective against Gram-positive bacteria, these micelles showed limited effectiveness against Gram-negative bacteria. To address this challenge, a random copolymer configuration was employed in place of a block copolymer, enhancing accessibility to the hydrophobic drugs and increasing capacity for membrane insertion and disruption [30]. Rod-shaped micelles are effective against *C. albicans*, a type of fungus that is difficult to eradicate due to its multilayered, thicker, and relatively less negatively charged cell walls [31,32]. There is some debate about whether the suitable polymer chains could exhibit increased activity when existing as free molecules in a solution, rather than supramolecular structures with reduced polymer chain mobility [33]. In systems relying on supramolecular structures, the reduced mobility may impede the cationic groups within the polymer, limiting their interaction with bacterial cell walls via electrostatic forces [26] or shielding the hydrophobic drug components within the core from the required disruptive interaction with bacterial membranes [34].

### 2.4. Polymer Vesicles

The “polymer vesicles”, also called “polymersomes”, are systems for biocide-releasing antimicrobial polymers. This class of artificial vesicles consists of hollow spheres (~50 nm to 5 µm in diameter) with a bilayer membrane that can be used for targeted and controlled drug delivery. The hollow cavity can be filled with both hydrophobic and hydrophilic drugs, and the bilayer membrane is made of amphiphilic synthetic linear diblock or triblock copolymers. One block is hydrophobic, and the other block or blocks are hydrophilic. Amphiphilic materials have both a water-loving polar moiety and a fat-loving nonpolar moiety. Polymers used for the hydrophilic blocks include polyethylene glycol (PEG) and polyethylene oxide (PEO), while the lyophilic or hydrophobic blocks can be made of polydimethylsiloxane (PDMS), poly(lactic acid), or poly(methyl methacrylate) (PMMA). Stronger interactions between the polymers and bacteria occurs when the polymers are self-assembled into vesicles which are structured as hollow spheres, compared to when polymer chains are unassembled. The polymersomes have three different regions available for functionalization: (i) the inner hydrophobic cavity, (ii) the polymer membrane, and (iii) the periphery [35,36]. Such polymer vesicles have been used for simultaneous antibacterial and anticancer medication [37].

### 2.5. Dendrimers

Dendrimers are unusual polymers that are multibranched with a spherical disposition and form another class of materials used as biocide releasing polymers. They can be designed with a core–shell architecture made of a hydrophobic core surrounded by repeating-unit branches with functionalized terminal groups [38,39]. Such a structure allows for stronger adsorption and binding to cell membranes compared to linear polymers [40]. Biocidal agents can be mobilized within the dendritic assembly by noncovalent encapsulation or by covalently binding to the terminal functional groups [40,41] followed by a targeted release [42,43,44]. The dendrimer loaded with the drug leads to the disruption and fragmentation of the bacterial membrane, subsequently resulting in the discharge of electrolytes and nucleic materials from the cell, ultimately leading to its death [41].

### 2.6. Polymeric Hydrogels

The “polymeric hydrogels” are 3D cross-linked polymers with hydrophilic groups that can be swollen by water. They can absorb more than 20% of their weight in water. Gelation (that is cross-linking in the presence of water) can be induced by a change in temperature, pH, or UV irradiation. They can have mechanical features and morphologies similar to natural tissues. They can be biocompatible; hence, hydrogels are also used for drug-delivery systems that are administered directly to the infected site. Their hydrophilic nature is advantageous for the solubility of antibiotics like ciprofloxacin, amoxicillin, or gentamicin [27]. Antimicrobial systems based on hydrogel polymers also overlap in their functionality with other categories of antimicrobial polymers which rely on using inorganic NPs like zinc oxide (ZnO) and metal NPs like silver (Ag) to kill microbes via multiple mechanisms [45,46]. Due to their water content, the hydrogels, whether loaded with antibiotics or NPs that kill microbes, cannot be used for shaping hard articles like a door handle.

### 2.7. Thermoplastics

Thermoplastics are polymers that can be melted and reshaped many times. When heated, they melt and harden when cooled. They can be either amorphous (the polymer chain structures are random and irregularly shaped) or semicrystalline (the polymer chain structures have a pattern and a definite shape that forms crystals) [47]. Homopolymers (chains that have one monomer type) and copolymers (chains with two or more bonded monomer types) are important when discussing polymers [48]. Other important terms are crystallization point T_c_ (change from liquid state to crystalline state), melting point T_m_ (change in physical state from solid to liquid), and glass transition point T_g_ (change from glassy to soft rubbery state) [49]. Common semicrystalline thermoplastics in food packaging and healthcare include polypropylene (PP), polyethylene (PE), and polyethylene terephthalate (PET), while polycarbonate (PC) is an amorphous type of thermoplastic.

Polypropylene (PP) is a semicrystalline product of propylene polymerization. It is created with a repeating propylene monomer (C_3_H_6_) that creates a long-chain (C_3_H_6_)_n_ polymer unit. It is translucent, semirigid, and resists heat and fatigue [50]. It also has good chemical resistance against bases and acids. It can be a homopolymer or copolymer (with ethylene), with the former having a higher strength-to-weight ratio, stiffness, and strength [50]. The melting point (T_m_) is 163.8 °C, it crystalizes at (T_c_) 123–129 °C, and it has a glass transition temperature (T_g_) of −25 °C [51]. This polymer is used for packaging and textiles [52]. Catalysts commonly used are metallocene or Ziegler–Natta. By 2022, the PP market stood at USD 126.75 billion [53].

Polyethylene (PE) is a product of ethylene polymerization and different types are industrially sold depending on the technology used. The chain structure is repeating ethylene (C_2_H_4_)_n_ molecules, which react in the presence of a catalyst, breaking the double bond and creating a chain [50]. There are three types of PE: High Density Polyethylene-HDPE (with organometallic catalyst), Linear Low-Density Polyethylene-LLDPE (typically made with the use of an alpha–olefin comonomer which also results in a low crystallinity), and Low Density Polyethylene-LDPE (made with organic peroxide initiator). HDPE is linear PE. LLDPE has short-chain branches from the incorporation of low amounts of butene or hexene comonomers [54]. LDPE has long chain branches and is produced via high-pressure polymerization of ethylene [50]. Mechanical features of LDPE include flexibility, transparency, and high-impact strength. LLDPE has a higher puncture and impact resistance and tensile strength than LDPE. HDPE is hard to semiflexible, resists moisture, and maintains high tensile properties. PE is generally inert and resists bases, acids, and oils [50]. They melt (T_m_) at 120–130 °C, crystallize (T_c_) at 110–120 °C, and have a glass transition point (T_g_) of −100 °C [51]. The polymer is used in carrying and fabricating materials due to its high chemical resistance, electrical insulation, transparency, and flexibility [55]. HDPE and LLDPE are made using the Phillips or Ziegler catalysts. By 2021, the polyethylene market stood at USD 102.49 billion [56].

Polyethylene terephthalate (PET) is a condensation polymerization product of terephthalic acid and ethylene glycol (EG) [57]. PET is processed using typical manufacturing methods such as extrusion, injection molding, and blow molding [58]. It can be semicrystalline or amorphous [50]. The structure consists of repeating C_10_H_8_O_4_ units. It has high Shore hardness and good tensile elongation, as well as excellent tensile strength and elasticity moduli [59]. Chemical features include resistance to low pHs [50]. It can be a homopolymer or copolymer. The glass transition point (T_g_) is between 69 °C and 85 °C, crystallization (T_c_) is at 170 °C, and the melting point (T_m_) is 260 °C [25]. It is used in textiles and packaging due to its high mechanical strength and durability. A commonly used catalyst is antimony trioxide [60]. By 2022, the PET market stood at USD 30.12 billion [56]. 

Polycarbonate (PC) is an amorphous polymer typically made from the condensation reaction of Phosgene (or carbonate) and Bisphenol A (PBA) polymerization. Phosgene and BPA are reacted in a methyl chloride solvent. The reaction is followed by centrifugation, filtering, concentrating, and drying the solution [61]. Catalysts, commonly tertiary amines, are used in the process. Chemical properties of PC include resistance to alkalis, acids, and water [50]. Mechanical features are high impact strength and stiffness. In practical use, PC is amorphous after extrusion with a glass transition temperature (T_g_) of 147 °C [62]. PC is used primarily in bulletproof windows due to its remarkable toughness, lightness, and almost unbreakable nature [63]. As of 2021, the PC market stood at USD 21.8 billion [64]. 

## 3. Antimicrobial Surface Modification

### 3.1. Design of Surfaces to Prevent Adhesion of Microbes

**Figure 2:**
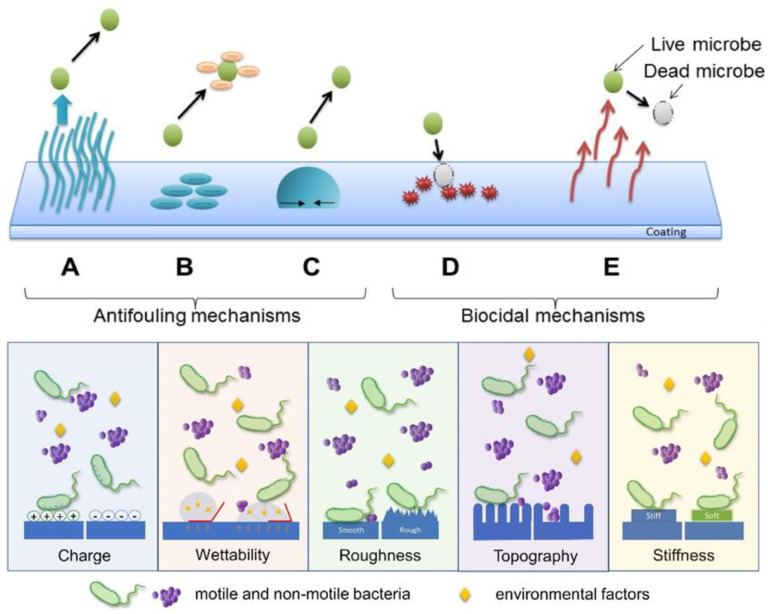
Instances of polymer-based antimicrobial mechanisms include the following: (A) steric repulsion, (B) electrostatic repulsion, (C) surfaces with low surface energy (demonstrating high contact angle), (D) release of biocides, and (E) direct contact-based microbial eradication (top), reprinted with permission from ref. [74], Elsevier license No: 5722911356576. Copyright (2016) Acta Materialia Inc. Diagram showing different surface characteristics that have a substantial impact on bacterial adhesion (bottom), reproduced from ref. [75], open access. Copyright (2021) Zheng, Bawazir, Dhall, Kim, He, Heo and Hwang.

A strategy to prevent microbes from depositing and proliferating on a surface is the use of microbial-repellent (antifouling) surfaces. They can be induced by coatings that aim to prevent the attachment of microbes and proteins on surfaces. In contrast to biocides, these surfaces do not actively kill microbes, but only prevent their attachment and growth on the surface where they could develop a biofilm. Figure 2, mechanism (A), (B), and (C), top and inset, illustrates the main principles used to prevent microbes from adhering to a surface, via alteration of the surface charge and chemistry, hydrophobicity, surface roughness, topography, or stiffness (Figure 2, bottom, inset). (Figure 2, (A), top, inset). Steric repulsion can be created, for instance, by having nano protrusions or cilia on the surfaces. Hydrophilic polymers, such as PEG oligomers use steric repulsion to repel microorganisms [65,66,67]. While a smooth surface favors adhesion, a surface with topography from the presence of nano protrusions prevents the clinging of microbes. Similarly, a soft surface favors attachment compared to a stiff surface. Figure 2, bottom, and inset, shows an antifouling mechanism induced via electrostatic repulsion. Zwitterionic polymers, which have both positive and negative charges, can be used for this purpose. Poly (2-methacryloyloxyethyl phosphorylcholine) (PMPC) [68], poly (sulfobetaine methacrylate) (PSBMA) [69,70], poly (carboxybetaine methacrylate) (PCBMA) [69], and polybetaines (PB) [71] are examples of materials that use electrostatic repulsion as the antiadhesion mechanism.

The method in Figure 2, (C), top, and inset, deploys surfaces with low energy where wetting is prevented. The structures required to prevent adhesion are, however, not easy to fabricate. Common thermoplastics, such as polystyrene (PS), polycarbonate (PC), and polyethylene (PE), have been used to fabricate textured antimicrobial surfaces [72]. They can be assembled into super hydrophobic micro/nanostructures which have low surface energy so that wetting is reduced (Figure 2, top, inset). When applied to such super hydrophobic surfaces, about 2% of the bacteria in a droplet adhered, and less than 0.1% persisted on the surface following rinsing [72]. Bacterial cells can colonize a super hydrophobic surface from a nanostructured topography only when air is entrapped between the water droplets, and the nano surface protrusions are excluded [73]. 

### 3.2. Killing Microbes by Making Biocidal Active Surfaces

While prevention of the adhesion and deposition of microbes on the surface is one design for antimicrobial polymers, another method is to fabricate surfaces that are biocidally active (Figure 2, (D) and (E), top, inset). Polymeric biocides and biocidal polymers are antimicrobial because of their intrinsic nature and/or due to biocidal groups and segments incorporated in the chain backbone and/or as sidechains [76,77,78]. A commonly employed biocidal component capable of being linked to different polymer chains is quaternary ammonium (QA) salt. QA salts are used as a disinfectant that kills bacteria, yeasts, and molds [79]. When a QA moiety has been attached to copolymers made of polymethylhydrosiloxane (PMHS) and polydimethylsiloxane (PDMS), antimicrobial films can be made. The QA salt moiety has also been attached to polyurethane (PU), and such PU can be used as an antimicrobial coating [78]. 

**Figure 3:**
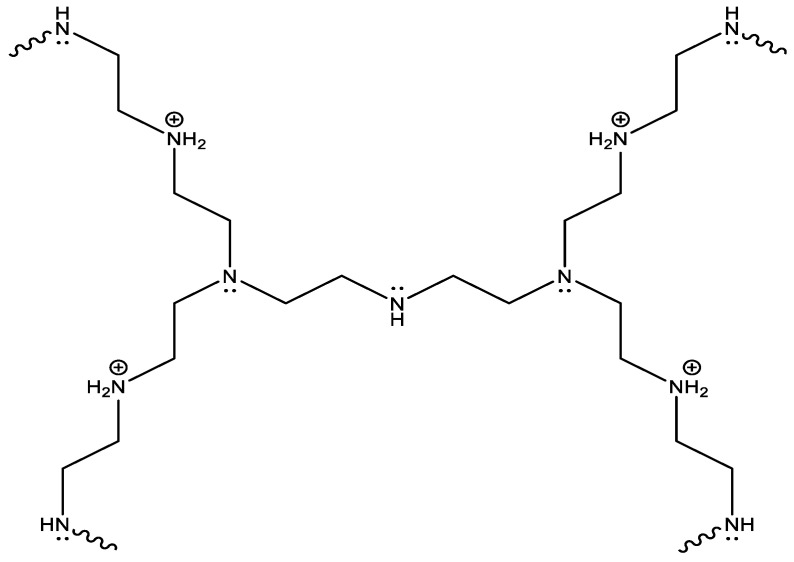
Chemical structure of polyethyleneimine (PEI) containing cationic moieties that are responsible for antimicrobial activity.

Alternatively, cationic polymers like polyethyleneimine (PEI) can be used to generate antimicrobial structures on surfaces. PEI polymers can be produced as a linear or branched polymer, or even as a dendrimer (Figure 3). The linear and branched polymer is soluble in hot water and melts at 73–75 °C and 59–60 °C, respectively [80,81]. The antimicrobial activity of PEIs derives from their cationic character, which can bind to the negatively charged membranes of the microbes that are attracted to it, leading to their puncture and cell lysis. Moreover, PEIs can undergo N-alkylation to incorporate quaternary amino functional groups, which, as previously noted, also possess antimicrobial properties.

N-halamine is another biocidal moiety capable of almost instant destruction of a broad spectrum of microbes [82,83]. N-halamine-containing polymers do not generate harmful substances or release halogens unless they are exposed to bacteria [84]. The synthesis of N-halamine-based polymers and graft copolymers can be economically achieved. While N-halamine polymeric surfaces are highly biocidal, their drawback is the loss of chlorine upon UV irradiation [85]. 

### 3.3. Polymeric Surface Structures Combining Antiadhesive and Biocidal Properties towards Microbes

It previously was mentioned that one strategy to fabricate antimicrobial polymers is to prevent deposition and adhesion of microbes on the surface, and another strategy is to have an active disintegration of microbes through biocidal groups and additives (or a combination of both). The first strategy is classified as biopassive, and the second is classified as bioactive. Some research has focused on combining the best approaches, which can drastically improve the overall antimicrobial properties of a material.

The chemistry of biopassive surfaces minimizes protein adsorption and maintains a surface charge to stop microbial proteins [86]. Creating these surfaces involves applying a coating and grafting [86]. Additives that can be used include antifouling agents such as polyethylene glycol (PEG), which forms dense layers over treated surfaces [87]. Important considerations include the type of surface, adhesion degree, and the longevity required [86]. 

Bioactive surfaces have immobilized bioactive molecules targeted at advancing or promoting specific interactions [88]. Their chemistry involves creating an apatite layer on a material in a substance by adding a bond through the solid material within it [89]. Additives that can be used include calcium phosphate and sodium titanates [89], depending on the material type and cell reaction.

The strategy of repel and kill microbes can involve conjugating antiadhesive polymers with biocidal moieties or polymers. Muszanska et al. demonstrated this approach with a triblock copolymer consisting of a core block of polypropylene oxide (PPG) and two side blocks of PEG (antiadhesive) [90]. In the PEG chain portion, Muszanska et al. added an antimicrobial enzyme (lysozyme) as a conjugate through covalent bonding. This particular triblock copolymer resulted in brush-like configurations consisting of one or two lysozyme molecules per chain of the triblock polymer. Surfaces treated with these brushes exhibited properties that prevented adhesion and inhibited microbial growth.

There are other combinations described for the simultaneous prevention of microbial deposition and biocidal activity. Antiadhesive PEG along with a cationic PC exhibited strong antibacterial and antiadhesive properties [91]. Its antiadhesive property was achieved through chain blocks leading to the assembly of particular structures while its biocidal property resulted from using specific mechanisms like cationic groups that are known to destroy cell walls.

**Figure 4:**
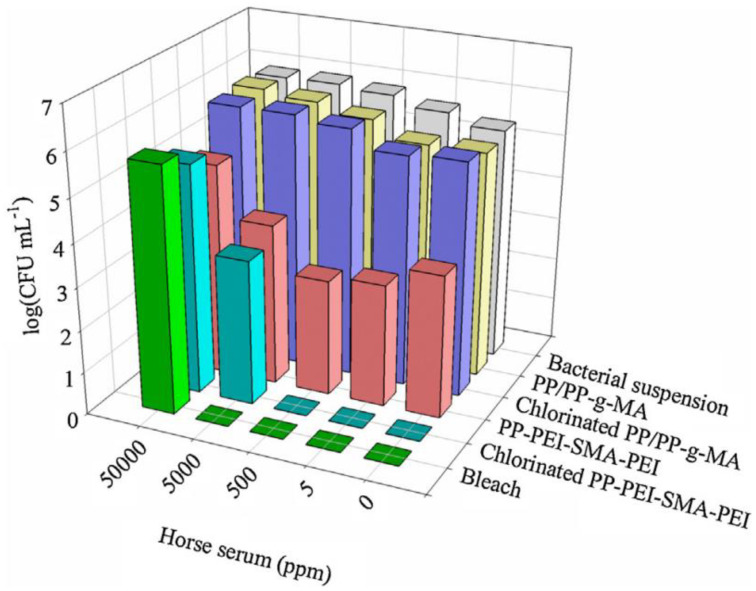
The antimicrobial characteristics of PEI/SMA/PEI systems, both chlorinated and nonchlorinated, compared to their counterparts. Reprinted with permission from ref. [92], Elsevier License No: 5722920789967. Copyright (2016) Elsevier B.V.

Such antiadhesive + biocidal polymers are often used as coatings. A conventional but modified plastic coupon was compression molded from PP/PP-graft-maleic anhydride (g-MA). A coating of branched polyethylene imine (PEI) and a styrene–maleic anhydride (SMA) copolymer was made into a block polymer with a PEI/SMA/PEI configuration, and this was spin-cast on the coupon from the PP/PP-graft-maleic anhydride [92]. The coating had 100 nm pores which were formed from hydrophobic styrene subunits that acted as an antiadhesion layer. The cationic primary amine groups had intrinsic biocidal properties and chlorinated N-halamine-based groups were attached. Figure 4 shows the bacterial count for PP/PP-g-MA, chlorinated PP/PP-g-MA, PP-PEI-SMA-PEI, and chlorinated PP-PEI-SMA-PEI. In the bar chart, bleach is shown as the benchmark for *E. coli* bacterial destruction. It can be seen that PP-PEI-SMA-PEI has activity against bacteria, but the chlorinated PP-PEI-SMA-PEI was also effective. The authors showed there was no *E. coli* adhesion on the PEI/SMA/PEI-coated PP surface [92]. 

### 3.4. Coating

Since only the surface of a material is in contact with microbes, a coating can be applied to confer antimicrobial properties that restrict the development of or kill microorganisms such as fungi [93]. Some coatings were developed to kill disease-causing microbes and pathogens through cellular membrane perturbation [94,95,96]. For example, antimicrobial polymer coatings are broadly utilized in dental implants. Antimicrobial surfaces also have an antibiofouling effect that resists the attachment of microbes to a surface. According to Chidanand et al. (2022), the global antimicrobial coating market was USD 3.7 billion in 2020 and is estimated to reach USD 11 billion by 2030 [97]. 

Alkarri et al. explores various fabrication methodologies aimed at creating antimicrobial polypropylene surfaces using both leachable and nonleachable antimicrobial agents. Through a comprehensive review of existing research and experimental approaches, the study evaluates the effectiveness of different techniques in incorporating antimicrobial properties into polypropylene materials. By examining both leachable agents, which release antimicrobial substances upon contact with microorganisms, and nonleachable agents, which are immobilized within the polymer matrix, the researchers assess the potential for long-term antimicrobial activity and durability. The findings highlight the importance of selecting appropriate fabrication methods based on the desired level of antimicrobial efficacy, durability, and compatibility with polypropylene substrates, providing valuable insights for the development of antimicrobial polypropylene surfaces with diverse applications in healthcare, consumer products, and other industries [98]. 

Avinash et al. discusses the development of functionalized nonwoven surfaces as a strategy to mitigate the spread of the COVID-19 pandemic. By incorporating antimicrobial agents and other functional coatings onto nonwoven materials commonly used in personal protective equipment (PPE), such as face masks and gowns, researchers aim to enhance their effectiveness in blocking viral transmission. Through various experiments and analyses, the study demonstrates the potential of these functionalized nonwoven surfaces to inhibit the viability and spread of viruses, including SARS-CoV-2. The findings suggest that the integration of antimicrobial and antiviral properties into nonwoven materials could play a crucial role in minimizing the transmission of infectious diseases, providing a valuable tool in the fight against the COVID-19 pandemic [99]. 

Chaitanya et al. investigates the enhancement of the antimicrobial properties of commercial face masks through the incorporation of colloidal silver nanoparticles (AgNPs). Through experimental analysis, the study demonstrates that the addition of AgNPs significantly improves the antimicrobial efficacy of the face masks, effectively inhibiting the growth of bacteria and potentially reducing the risk of infection transmission. The findings suggest that the incorporation of colloidal silver nanoparticles into face masks could offer an effective strategy to enhance their protective capabilities against microbial pathogens. Further research is needed to optimize the formulation and application of AgNP-based treatments in face masks, but initial results indicate promising potential for improving the antimicrobial response of commercial face masks [100]. 

Struszczyk et al. presents preliminary findings on the potential antimicrobial behavior of nonwovens coated with chitosan. Through experimental analysis, the study investigates the effectiveness of chitosan-coated nonwovens against microbial growth. Results suggest promising antimicrobial properties, indicating a potential application for these coated nonwovens in various sectors where microbial contamination is a concern. Further research is warranted to fully explore the extent of antimicrobial activity and to optimize coating techniques for enhanced efficacy, but initial findings highlight the viability of chitosan-coated nonwovens as a potential solution for combating microbial growth on surfaces [101]. 

Novikova et al. explores the modification of cotton fabrics with molybdenum nanoclusters to enhance their ability for photodynamic inactivation of bacteria and viruses. Through a series of experiments, the researchers demonstrated that the molybdenum nanoclusters significantly improved the fabrics’ antimicrobial properties under visible light irradiation. The modified cotton fabrics exhibited effective photodynamic inactivation of both Gram-positive and Gram-negative bacteria, as well as viruses. Furthermore, the study highlighted the potential of these molybdenum-modified fabrics for various applications, including medical textiles and personal protective equipment, due to their enhanced antimicrobial activity [102]. 

## 4. Antimicrobial Polymers

### 4.1. Antimicrobial Polymer Blends

**Figure 5:**
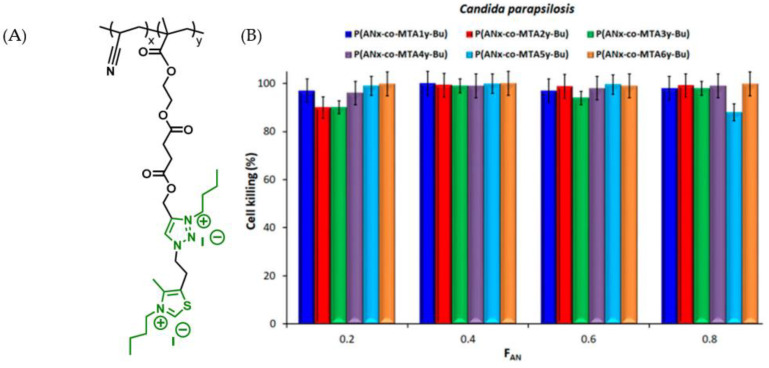
The chemical configuration of quaternized P(AN-co-MTA) copolymers originating from acrylonitrile (**A**). The degree of cell elimination against the *C. paraplosis* microorganism upon exposure to antimicrobial films (**B**). Reproduced from ref. [103], open access.

Blending polymers possessing antiadhesive or antimicrobial properties can create peculiar nanostructures and chain conformations that amplify these two effects to prevent microbial growth. An example is to blend an inert polymer with a copolymer derived from it and decorate the latter with antimicrobial moieties. A specific case utilized a blend of polyacrylonitrile (PAN) and antimicrobial methacrylic copolymers with attached cationic moieties (1,3-thiazolium and 1,2,3-thiazolium side-chain groups), P(AN-co-MTA) (Figure 5A).

The biocidal cell-killing efficiency was significantly improved, to almost 100%, through the increase in the positive charge density (Figure 5B). An additional example of a surface possessing biocidal property involved a polymer combination containing polystyrene (normally inactive) and block copolymers consisting of polystyrene combined with an antimicrobial block called poly(4-(1-(2-(4-methylthiazol-5-yl)ethyl)-1H-1,2,3-triazol-4-yl)butyl methacrylate), which was decorated with methyl or butyl groups (PS-b-PTTBM). This blend yielded a highly positively charged material.

Likewise, one way to influence the adhesive properties of the polymer system is to blend a protein-repellent polymer, such as PEG, with other inert polymers, such as polyacrylic acid (PAA). These adhesive properties were achieved by developing brush-like films made with optimized ratios and molar masses of PEG and PPA [104]. 

Blending polymers that repel microbes (biopassive) with those possessing biocidal properties allows for the creation of polymeric structures that demonstrate both active microbe-killing capabilities (bioactive) and resistance to biological elements [105]. Additionally, combining one biocidal polymer with another enhances synergistic biocidal properties, significantly intensifying microbe eradication.

In all the methods above, some of the polymers with biocidal moieties are either not scalable to commercial production due to the cost, or the antimicrobial system can only be applied as a coating. Another limitation is that many of the inherently biocidal polymers would not be extrudable or injection moldable, as they contain moieties that make the polymer dissolve or swell in water. The mechanical resilience and wear of these coatings and their moisture-induced tackiness have not been studied or reported. However, they might create issues for practical use.

### 4.2. Leachable and Nonleachable Antimicrobial Agents

**Figure 6:**
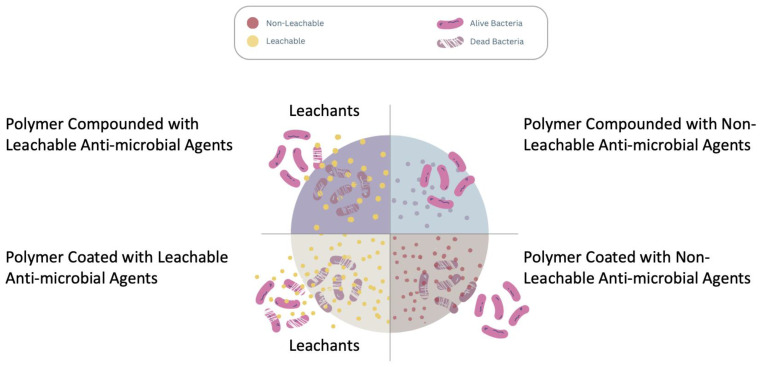
The mode of action for leaching and non-leaching antimicrobial agents when used in plastics through melt-compounding (left) and via thermal embossing (right). Reprinted with permission from ref. [98], John Wiley and Sons License No: 5722850541962. Copyright (2023) Wiley Periodicals LLC.

Leachable antimicrobial agents are substances embedded into plastic articles that release antimicrobial features via diffusing or leaching, thereby stopping microbial growth in the surrounding environment [106]. The primary feature is their ability to be physically embedded in a matrix. One substance, silver nano particles (Ag NPs), has been extensively studied. For centuries, silver has served as a biocide. Upon exposure to moisture, an electrochemical process occurs, releasing silver ions. These ions penetrate microbes, incapacitating their ability to function, proliferate, or reproduce. Advantages include continued protection and functionality against various microorganisms [107,108]. A drawback is the loss of antimicrobial properties over time since leaching will cause the depletion of the agent, especially in applications that come in contact with water. In addition, the use of nanosilver is controversial. For instance, some research indicates that prolonged exposure to silver ions may adversely affect human health. Also, waste management is a concern if the disposed silver spills into the surrounding environment. [109,110]. Figure 6 shows a schematic diagram depicting the modes of action for “leaching” and “non-leaching” biocides.

To overcome these issues, non-leachable antimicrobial agents were sought. These agents, that cannot pose a contamination risk to the surrounding environment, may function as coatings that remain on the surface of the treated article. Microbial killing therefore occurs through direct interaction with these substances on the surface of treated articles. The antimicrobial activity is thus confined to the surface of treated articles and does not leach or diffuse into the surrounding environment [111]. This effect can be achieved through chemical embedding, and coatings are the primary implementation. Examples include organo-silanes [112]. Advantages include long-lasting protection and a low risk of leaking to the environment, and coating can be applied after the article has been manufactured. Nonleachable antimicrobial agents are inexpensive and could be applied as a coating for plastics. The drawback is a limited range of antimicrobial protection when used in melt compounding [106]. 

Inorganic antimicrobial agents

Ag NPs

Ag NPs are made up of nanoscale Ag. Applications include sterilization of medical and consumer products such as textiles and refrigerator surfaces [113]. Features include that they are oligodynamic, have a high surface-area-to-volume ratio, and possibly can be synthesized into multiple shapes and sizes [114]. They are mostly insoluble in water [115]. The ionic radius of Ag ions is 126 pm [116]. Toxicity is dependent on particle size. They work by disrupting cell walls and cytoplasmic membranes, denaturing ribosomes, stopping the production of adenosine triphosphate, eliciting the production of reactive oxygen species (ROS), membrane disruption, DNA replication interference, and denaturing and perforating membranes [117]. However, because Ag NPs can easily enter human skin, [118] toxicity is an important consideration [118]. Still, the FDA has approved some Ag-based NPs for wound dressing and antimicrobial products [119]. 

Cu NPs

Nanoscale copper particles are used for antimicrobial purposes and porosity enhancement. Features include robust antimicrobial activity and a high surface-area-to-volume ratio. They are not soluble in water. Cu has an ionic radius of 57 pm [120]. Cu NPs can be rated as class 3, which is moderately toxic [121]. They work by generating reactive oxygen species (ROS), which interferes with cellular function and leads to DNA damage [122]. The FDA approves several Cu-based compounds for particular purposes.

CuO NPs

Copper oxide nanoparticles are used as antimicrobial agents in industrial, biomedical, and agricultural fields [123]. They have a brownish-black color, have antimicrobial properties that are effective against a broad range of pathogens and bacteria, are relatively inexpensive, have a comparatively longer shelf life, and have a significant surface-area-to-volume ratio [124]. They are slightly soluble in water but only up to 37.1–100.1 mg·kg^−1^ [125]. Cu^++^ ions have an ionic radius of 73 pm [126]. They work by creating ROS that damage biomolecules and cell membranes [127]. However, they can be toxic to living organisms, causing ROS generation, cytotoxicity, oxidative stress, immunotoxicity, and genotoxicity [128]. Information on FDA approval is not available.

CaO NPs

Calcium oxide NPs are used in water purification, catalysis, antibacterial agents, adsorption, and purifying vehicle exhaust [129]. Properties include their use as a white powder, spherical shape, and high adsorption efficiency [130]. They are slightly soluble in water [131]. Calcium ions (Ca^++^) have an ionic radius of 99 pm [132]. The primary methods of action are ROS on its surface and the alkaline effect [133]. They are generally safe for humans in low concentrations but can cause respiratory issues when inhaled. These NPs are not approved by the FDA, but calcium oxide is recognized as a safe substance that can be used in plant and animal products [134]. 

MgO NPs

MgO NPs have shown antimicrobial activity against different fungi and bacteria including antibiotic-resistant strains. Uses include water treatment systems, food packaging, and healthcare products [135]. They have enhanced stability, a high surface area, and potent antimicrobial characteristics. They do not fully dissolve in water but are slightly soluble at high pH levels (1.71 = mg L^−1^ at pH = 6.69) [136]. The ionic radius of Mg^++^ is 72 pm [137]. They can disrupt cellular activity and release ROS. They are generally considered safe (they are used as antacids), although long-term exposure may lead to lung irritation or injury [138]. They have been approved by the FDA as a safe and functional antibacterial agent [139]. 

Mg(OH)_2_ NPs

Mg(OH)_2_ NPs consist of inorganic compounds used as antiacids. They can act against bacteria, fungi, and viruses including COVID-19. They are applied in medical devices, wound healing, and drug delivery systems. They have a large surface area, are biocompatible, and have good stability [140]. They are sparingly soluble in water and able to form colloidal suspensions. The ionic radius of Mg^++^ is 72 pm [137]. The mode of action involves activating ROS [141]. They are generally considered safe, although high concentrations can cause gastrointestinal complications. They are FDA approved.

Cu-Infused Mg(OH)_2_ NPs

They contain magnesium hydroxide incorporated with copper ions in their structure. They have enhanced antimicrobial activity due to the Cu ions that possess inherent antimicrobial properties. They are stable and biocompatible with Mg(OH)_2_. Mg(OH)_2_-based NPs are sparingly soluble in water at 0.00122 g/100 mL [142]. The ionic radius of the Cu ion is 73 pm, while that of Mg^+^ is 72 pm [143]. Cu^+^ is toxic at higher concentrations [144]. The mechanism of action is the release of Cu ions, which can cause oxidative damage to microbial cells [145]. Biomedical applications include wound dressings and implants. They are not currently approved as safe by the FDA [146]. 

CuCl_2_ NPs

CuCl_2_ NPs are antimicrobial agents consisting of copper and chlorine ions. They are primarily utilized in water treatment, surface coatings, and medical devices. Unique features include a high surface-area-to-volume ratio, increased reactivity, and enhanced stability. They are water-soluble and can be applied in aqueous environments [147]. The ionic radius for Cu ions is around 73 pm, while that for chlorine ions is 181 pm [148]. They are toxic to human cells, though toxicity is dependent on concentration [149]. The mode of action comprises cell membrane disruption, causing intracellular components to leak [150]. They can also cause ROS, causing damage to various cellular components. They are not FDA approved.

TiO_2_ NPs

TiO_2_ NPs are made up of nanoscale titanium dioxide. They restrict bacterial growth, have a high refractive index of 2.4, are transparent, and exhibit strong absorption of ultraviolet energy [151]. Applications include toothpaste, paper, plastics, drugs, textiles, and cosmetics [152]. They are generally insoluble and resist dissolution [153]. They are potentially toxic due to their potential to induce liver and brain lesions in humans and lung tumors in rats [154]. Mode of action includes ROS, lipid peroxidation, cell wall damage, and attachment to biological macromolecules and intracellular organelles [153]. TiO_2_ is approved by the FDA for use in food and drug-related products [153]. 

ZnO NPs

ZnO NPs are made of zinc oxide. Its properties include that it is a white powder with a tetrahedral shape, is thermochromic, and is insoluble in water (may dissolve between 0.3 and 3.6 mg/L) [155,156]. The material is insoluble in water. The ionic radius of Zn^++^ is 79.6 pm [157]. Toxicity of ZnO NPs is unlikely in humans because they rarely or minimally penetrate the stratum corneum and other skin layers [158]. According to Mendes et al. (2022), ZnO NPs are multitarget compounds that impact numerous microbe structures [159]. However, their primary mechanism of action is by damaging the cytoplasmic membranes. They produce ROS, increasing membrane lipid peroxidation [160]. They are primarily used to coat, paint, and in cosmetics. However, they can induce oxidative stress and trigger apoptosis [161]. The FDA approved these NPs due to safety and nontoxicity in low concentrations [162]. 

GO NPs

GO NPs are made of graphene oxide. GO agents are primarily used in cellular imaging, drug delivery, and cancer treatment [163]. Despite having a nanoscale size, the oxide induces oxidative stress and interacts with the cell membrane, making it toxic [164]. It is hydrophilic due to ketones, hydroxyl groups, and carboxylic acids on the graphene. It has a high specific surface area of about 890 m^2^g^−1^. It is also a semiconductor [164]. Since it is hydrophilic, the NPs usually dissolve in water, with the highest solubility of 6.6 µg/mL in deionized water [165,166]. At particular concentrations, GO can be toxic to species such as earthworms and zebrafish [164,167]. The mode of action of the material remains unclear despite extensive research. Suggested modes include oxidative stress generation [168]. The FDA has not approved the material as an antimicrobial agent, but there are approvals for cancer drug delivery systems such as PEGylated liposomal doxorubicin [169]. 

Shape and size of NPs

The shape and size of NPs play a substantial role in their antimicrobial activity. Smaller NPs have a higher surface area relative to their volume [170]. This improves the interaction with microbes, providing better antimicrobial protection since microbes are more likely to be inhibited [170]. They are also more capable of penetrating microbes especially if the NPs have sharper edges. Smaller NPs which can create reactive oxygen species (ROS) allow higher generation of ROS, improving the ability to kill microbes [171]. The shape also has an impact [171]. Shapes such as nanotubes cause physical damage to microbes by piercing membranes leading to deterioration and death [172]. 

Soluble and nonsoluble antimicrobial agents

Soluble antimicrobial agents, such as antiseptics, antiparasitics, and antibiotics, dissolve in water [173]. Costs differ based on type, concentration, and quantity but are generally affordable. For example, Walmart sells a 32 oz. bottle of Equate 91% isopropyl alcohol antiseptic for USD 3.98 and Equate 32 oz. 70% isopropyl antiseptic for USD 2.96 [174]. Advantages include proven effectiveness, availability in numerous formulations, and effectiveness against bacteria, viruses, and fungi. Cons include side effects and resistance, especially for bacteria.

Nonsoluble agents do not need to dissolve in a liquid to be applied to a surface or object. These include metal surfaces, polymers, and metallic NPs [175]. Advantages include lasting longer and killing more types of microbes [175]. Cons include environmental risks and costs. Because they do not diffuse from the point of application there is no problem with creating a ‘kill zone’ in the proximity of the treated object, and, as noted earlier, the concentration of NP on a treated article does not diminish due to diffusion away from its point of application.

## 5. Polymer Nanocomposites with Biocidal Metals and Inorganic Nanocrystals

In this process, an extrinsic biocidal additive is blended with an inert polymer or with a biocidal polymer. Certain metals and inorganic crystals (often metal oxides and hydroxides) show biocidal properties. As mentioned above, well-known examples of the former are Nano Ag and Nano Cu as well as carbon-based materials like carbon nanotubes and graphene. As previously mentioned, examples of inorganic crystals that show biocidal properties include copper oxide (CuO), titanium dioxide (TiO_2_), magnesium oxide (MgO), magnesium hydroxide (Mg(OH)_2_), and zinc oxide (ZnO). One approach to fabricate nanocomposites with these materials is to combine them with polymeric biocides. For example, biocidal Ag NPs [162], Cu NPs [163], or carbon nanostructures [164] can be added to polymeric biocides such as PEG [165], PEI [166], zwitterionic PCBMA [167], or cationic poly(2-(tert-butylaminoethyl) methacrylate) (PTBAM) [168], to develop highly biocidal nanocomposites (Table 1).

## 6. Attraction between NPs and Microorganisms due to Electrostatic Charges

Charge attraction between NPs and microorganisms serves a substantial role in antimicrobial activity [187]. NPs are typically positively charged [25], while the microorganisms have a negatively charged phospholipid membrane. When they interact, microorganisms are inhibited and die from different modes of actions, such as the creation of ROS, electrostatic interaction, intercutting of membranes, and microbe resistance reduction, depending on the NPs nature [188]. 

## 7. Mode of Mechanisms of Antimicrobial NPs

**Figure 7:**
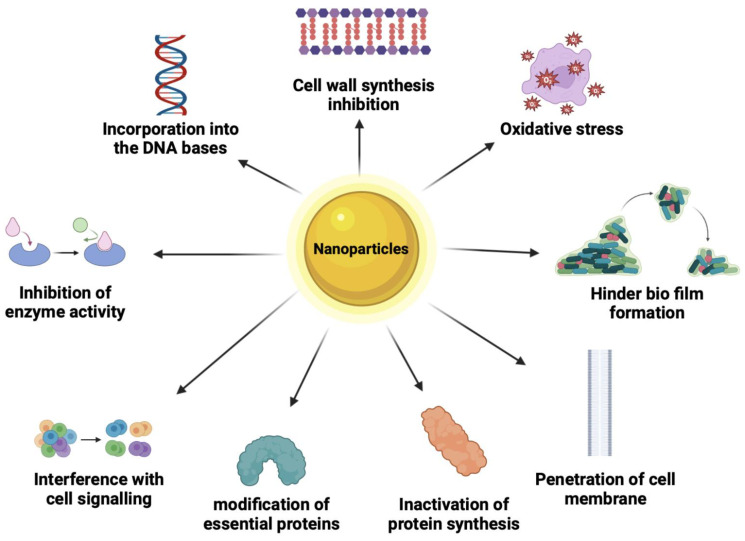
Summaries of the multiple mechanisms by which biocidal nanoparticles can damage bacterial cells. One or more mechanisms may operate with a given NP. Modified from ref. [189]. Copyright (2014) American Scientific Publishers.

Nine different modes of action have been established to describe how antimicrobial NPs can kill microbes, as illustrated in (Figure 7). These modes of action will be described below. A particular emphasis on inorganic NPs’ mode of action is also described.

### 7.1. Cell Wall Synthesis Inhibition

The cell wall of a bacterial cell includes the membrane and the peptidoglycan layer, which is composed of long sugar polymers. The role of this peptidoglycan on the cell is to protect it from stress and maintain its integrity. The peptides within peptidoglycan are interconnected through cross-linking facilitated by trans glycosidase, aiding in their development. Further linking of glycine residues contributes to the durability of the bacterial cell wall [190]. When NPs or beta glycopeptides are present, they specifically inhibit cell wall synthesis by impeding the cross-linking or assembly of peptidoglycan.

### 7.2. Oxidative Stress

Aerobic bacteria rely on oxygen for the oxidation of nutrients to generate energy. Reactive oxygen species (ROS) induce bacterial death by directly damaging lipids and proteins through oxidation. ROS can trigger nonoxidative mechanisms like autophagy, leading to bacterial demise. Moreover, an imbalance in ROS levels creates oxidative stress, causing an accumulation of uncontrolled hydroxyl radicals [191]. These radicals, in turn, degrade nucleic acids, proteins, and carbohydrates, culminating in cell death.

### 7.3. Protein Synthesis Inhibition

Proteins are organic molecules in bacterial cells that have both structural and enzymatic roles [190]. NPs can disrupt the structure of protein molecules and thus their function in cell architecture and metabolism.

### 7.4. Inhibition of Enzyme Activity

Enzymes play crucial roles in numerous cellular functions. For instance, transpeptidase catalyzes the final stage of cell wall biosynthesis [192]. When antimicrobial NPs attach to the active sites of these enzymes, they diminish the compatibility between the enzyme and its substrate. Consequently, this inhibition prevents the enzymes from functioning, impeding essential cellular processes within the cell.

### 7.5. Hinder Biofilm Formation

Biofilms refer to intricate layers of matrices surrounding bacteria, aiding bacteria in their survival in challenging or adverse environments [193]. Microbial cells that attach to a surface in a moist environment can survive and proliferate. When the microbial cell number increases, it leads to a biofilm formation, a polysaccharide matrix with embedded cells. Biofilms allow microbial cells to survive under harsh conditions and are 1000 times less susceptible to most biocides. Therefore, a biofilm protects the bacteria from any form of stress. Toxins excreted from biofilms spread pathogenic and resilient infections and can lead to multidrug-resistant bacterial strains. NPs target any of the three stages of biofilm formation which are adhesion, maturation, and dispersal of the biofilm. For example, a NP can target adhesion; thus, the bacteria will not have the capacity to attach to a surface.

### 7.6. Interference with Cell Signaling

Cell-to-cell communication is important in coordinating the behavior of a group of bacterial cells. These behaviors within a set of bacterial cells are important in building an antibiotic tolerance. Antimicrobial agents can target cell-to-cell communication, hindering the group from building resistance to antibiotics [194]. 

### 7.7. Modification of Essential Proteins

Cells rely on some essential proteins to carry out metabolism and other processes. By modifying essential proteins such as ribosomal proteins, enzymes, or membrane proteins a bacterial cell may lose the capacity to carry out normal metabolic processes [195]. Therefore, by altering the configuration of the proteins NPs can inactivate the bacterial cells’ functions.

### 7.8. Penetration of Cell Membrane

NPs affect the cell membrane by binding to it through electrostatic forms [196]. The results are changes in the protective qualities of the cell membrane, loss of membrane polarity, and even disintegration of the cell membrane. For example, Ag NPs cause pits on the cell membrane, causing increased permeability [196]. Consequently, increased permeability causes an imbalanced transport of ions in and out of the cell, causing cell death.

### 7.9. Incorporation into the Nucleic Bases

NPs can be incorporated into the nucleic bases of cells, changing their configuration [197]. Nucleic acids are responsible for the coding of genetic information. Therefore, when NPs are incorporated into the bacterial cell, DNA cannot be transcribed which then inhibits protein synthesis; hence, the cells lose functionality.

### 7.10. Mode of Action for Inorganic NPs

As mentioned above, Ag exhibits biocidal properties against various bacteria, fungi, and viruses because of its diverse mechanisms of action. This includes the release of Ag^+^ ions when in contact with water. The liberation of Ag^+^ ions is influenced by various factors, including the dimensions and configuration of the NPs. Smaller NPs would have a large surface-area-to-volume ratio and hence release more ions. Ag ions interact with -SH groups on the bacterial cell surface because there is a large number of sulphur-containing proteins in the bacterial cell membrane [198]. The released Ag^+^ ions from Ag NPs can engage with the phosphorus components in DNA, resulting in the deactivation of DNA replication [198]. Thus, silver ions (Ag^+^) engage in detrimental interactions with proteins found in the microbial cell wall and membrane, resulting in disruptions to cellular transport systems, imbalances in electrolytes, membrane puncturing, loss of cytoplasmic organelles, and modification of bacterial cell division, ultimately resulting in cell demise [199]. They are active against both Gram-positive and Gram-negative bacteria [45,46]. 

Another antibacterial mechanism of Ag^+^ is reactive oxygen species (ROS) generation. ROS are highly reactive molecules and radicals formed from O_2_ such as peroxides, superoxide, hydroxyl radical, singlet oxygen, and alpha oxygen. Table 2 shows some examples of ROS. While ROS are used as cell signaling molecules for normal biological processes, the generation of too many ROS can damage multiple cellular organelles and processes. The ROS created by Ag^+^ induce oxidative stress in bacterial cells [198]. 

It is important to consider that while ROS generation by NPs may be effective against microbes, excessive amounts of ROS can result in the same damage to human cells.

Ag NPs have been thus added to inert as well as biocidal active polymers. For example, they have been added in inert PMMA and in the polymeric biocide PTBAM (cationic poly(2-(tert-butylaminoethyl) methacrylate) to form nanofiber composites [177]. Both these Ag/nanofiber-based composites exhibited great antimicrobial performance against *Escherichia coli* (*E. coli*) and *Staphylococcus aureus* (*S. aureus*), but the PTBAM/Ag nanocomposite was naturally better as the matrix itself was a polymeric biocide. Ag has been added in zwitterionic PCBMA and has also been used to make polylactide (PLA)/PEG/Ag nanocomposites. Thus, the Ag reinforced the antimicrobial properties of the polymeric biocide PCBMA [179], and acted cooperatively with the antiadhesive properties of PEG [180] polymers to form an effective antimicrobial nanocomposite. Like nano Ag, nano Cu also has strong antimicrobial properties, and thus has been used with polymeric biocides and inert polymers [200]. Cu also kills bacteria through multiple mechanisms, including the release of toxic Cu ions [201] and the inhibition of biofilm formation [202]. Details about these mechanisms can be found in the review by Tamayo et al. [200]. Examples of the reinforcement of antimicrobial properties of polymers containing biocidal Cu include PEI/Cu and nanocomposites of pectin with Cu^++^ incorporated into PEI.

Among the inorganic crystals, TiO_2_ disrupts bacterial cell membranes. It eliminates bacteria by creating reactive oxygen species (ROS) upon exposure to light. ZnO demonstrates antibacterial effects with minimal toxicity to mammalian cells. Its mode of action involves causing damage to the lipids and proteins found in the bacterial cell membrane and triggering ROS production. ZnO NPs exhibit efficacy against bacterial spores resistant to high temperatures and high pressures [45,46]. 

Motoike et al. [203]. compared the activity of dolomite powder, CaO, MgO, and Mg(OH)_2_ against the avian influenza virus strain A/whistling swan/Shimane/499/83 (H_5_N_3_). They studied the effect of heating the powder and found a relation between the crystal sizes of the minerals and the antiviral activity. Dolomite is a natural mineral composed of calcium and magnesium carbonates (CaMg(CO_3_)_2_) and it can be decomposed to MgO and CaO.

CaMg(CO_3_)_2_ → MgO + CaCO_3_ + CO_2_ at 750–800 °C (1)

CaCO_3_ → CaO + CO_2_ at 900–1000 °C (2)

Both MgO and CaO have strong activity against viruses. CaO and MgO, obtained via the thermal decomposition of dolomite above 800 °C, had strong antiviral activity, but the effect was weakened when the temperature reached beyond 1400 °C; the effectiveness of CaO surpassed that of MgO but quickly hydrated in the presence of water which reduced its antiviral activity. Conversely, the hydration process of MgO occurred more slowly under identical conditions. When comparing MgO and Mg(OH)_2_ separately, it was observed that MgO exhibited a greater antiviral effect than Mg(OH)_2_.

**Figure 8:**
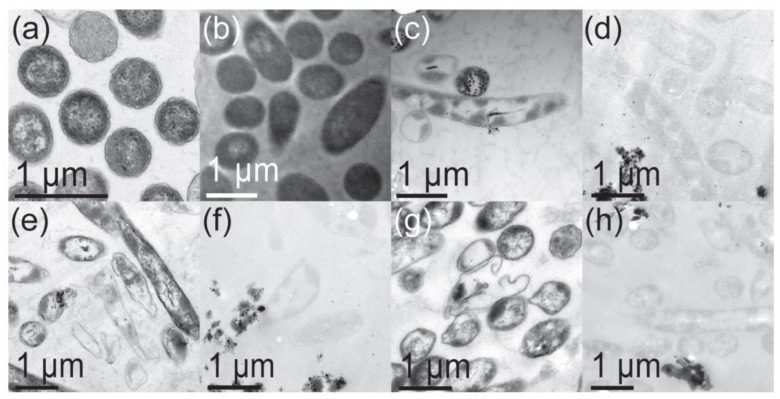
Transmission electron microscopy (TEM) images depict *E. coli* bacteria post UV and/or MgO nanoparticle exposure: (**a**,**b**) control images with and without staining; (**c**,**d**) images of 1-MgO with and without staining; (**e**,**f**) images of 1A-MgO with and without staining; and (**g**,**h**) images of 2-MgO with and without staining. Reprinted with permission from ref. [204], John Wiley and Sons License No. 5723251223710. Copyright (2013) Wiley-VCH Verlag GmbH & Co. KGaA, Weinheim.

MgO NPs create ROS, but they also release some Mg^++^ cations; however, Mg^++^ does not have the same destructive impact as Zn^++^ [204]. Leung et al. investigated the destruction of *E. coli* bacterium by MgO NPs and found that there were non-ROS mechanisms also. Figure 8 displays Leung et al.’s TEM images of *E. coli* bacteria, which show cell membrane damage, after treatment with three different MgO NPs. There was no evidence of MgO NPs penetration into the cells. As these authors did not find evidence of lipid peroxidation (oxidative stress), they concluded that non-ROS-based mechanisms exist for this combination (MgO and *E. coli*). They hypothesized that the damage was due to a combination of the attachment of the NPs to the membrane and the effects of pH change and Mg^++^ release.

**Figure 9:**
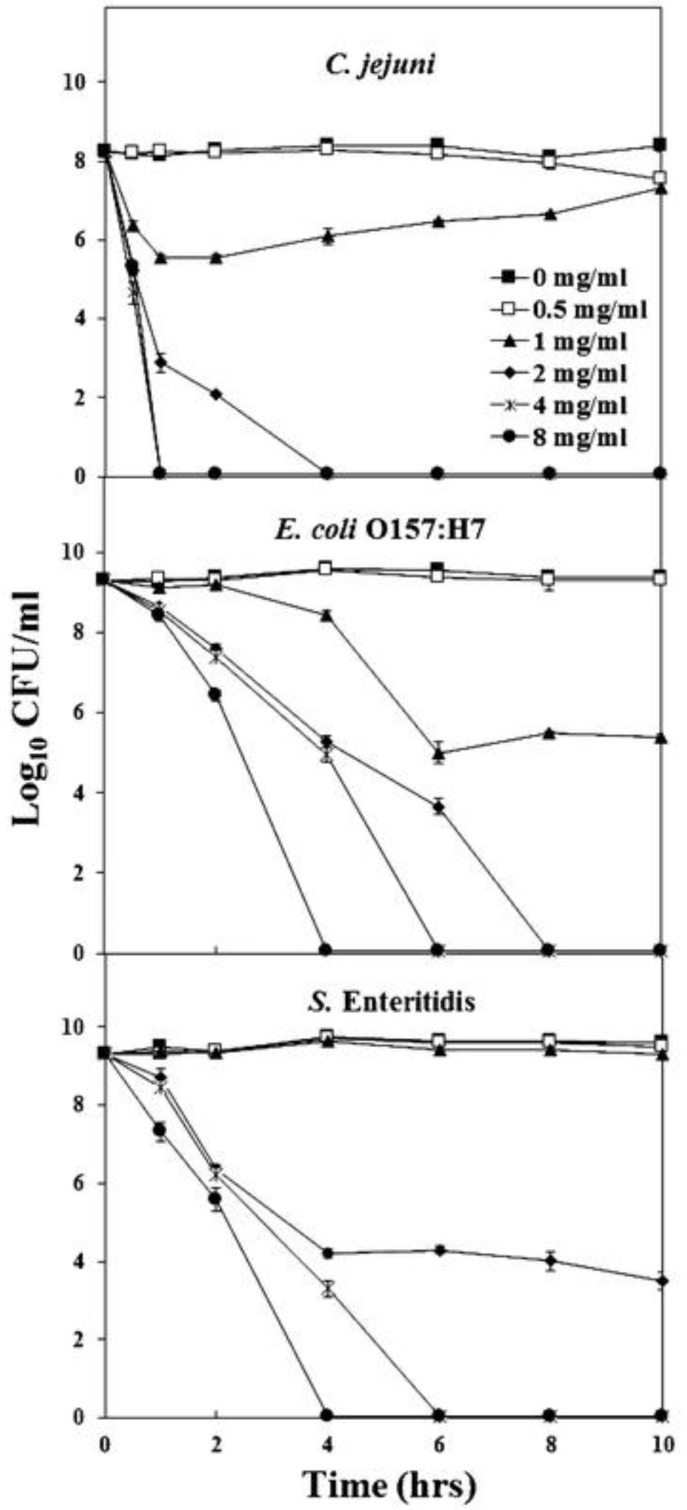
The antimicrobial impact of MgO nanoparticles against *C. jejuni*, *E. coli* O157:H7, and *S.* Enteritidis was evaluated. Different concentrations of nanoparticles were administered to approximately 108 CFU/mL of *C. jejuni* and 109 CFU/mL of either *E. coli* O157:H7 or *S.* Enteritidis. Subsequently, at various intervals following treatment, viable cell counts were determined by culturing bacterial colonies on MH agar plates. Each CFU/mL value represents the average from six separate replicates. Reproduced from ref. [205], open access. Copyright (2016) Yiping He, Shakuntala Ingudam, Sue Reed, Andrew Gehring, Terence P. Strobaugh Jr, and Peter Irwin.

The antimicrobial activity of MgO NPs (Figure 9) against a variety of microorganisms, such as *C. jejuni*, *E. coli*, and *S.* Enteritidis, was studied at six different time intervals (0, 2, 4, 6, 8, and 10 h).

He et al. [205]. reported the effect of ZnO on the three most common foodborne pathogens *Campylobacter jejuni* (*C. jejuni*), *E. coli* O157:H7, and *Salmonella*. These bacteria reside in the intestinal tracts of birds (e.g., chicken) and cattle [4,5,206]. These pathogens can be transferred to food throughout the stages of harvesting, processing, distribution, and preparation. Contamination with these bacteria has been found in meat, dairy products, and other foods. In the study, a suspension of ZnO nanocrystals with a redox dye that changed color was added to bacterial broths of these three pathogens. Metabolically active cells reduced the nonfluorescent blue resazurin dye to fluorescent red resorufin. Since inactive cells do not undergo resazurin reduction, no color change from blue to red would indicate cell death.

**Figure 10:**
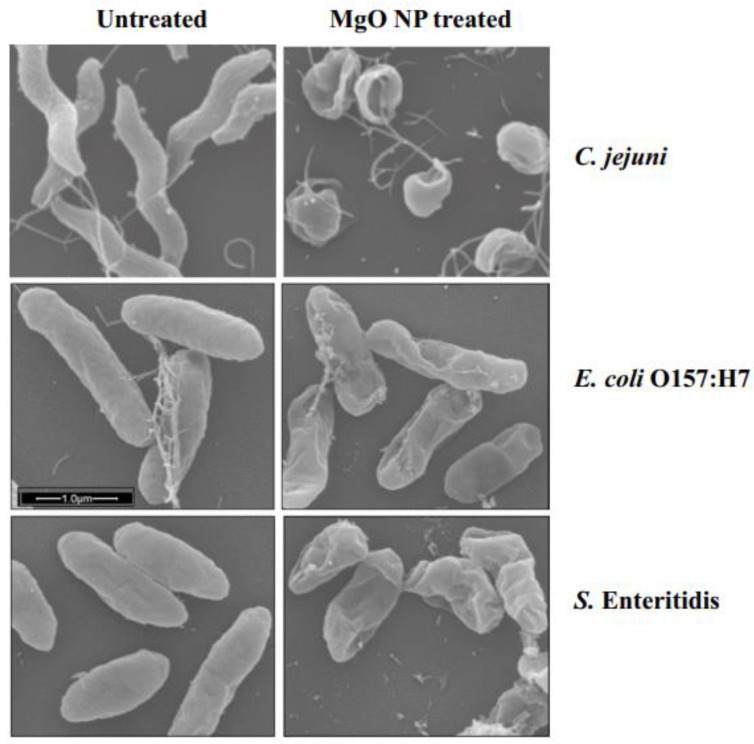
Scanning electron micrographs depict the bacterial cells of *C. jejuni*, *E. coli* O157:H7, and *S.* Enteritidis. SEM images were captured from treated bacterial cells of *C. jejuni*, *E. coli* O157:H7, and *S.* Enteritidis (right panel) subjected to 2 mg/mL MgO nanoparticles for 8 h. The control cells (left panel) were incubated under identical conditions without the addition of nanoparticles. Reproduced from ref. [205], open access. Copyright (2016) Yiping He, Shakuntala Ingudam, Sue Reed, Andrew Gehring, Terence P. Strobaugh Jr, and Peter Irwin.

He et al.’s [205]. SEM images (Figure 10) showed the untreated cells of all three bacteria had intact and smooth surfaces. The cells treated with sublethal doses of MgO displayed deep craters on their membrane surface. The shortening of the cells suggested there could be some leakage of the cellular contents caused by the treatment. These authors showed that oxidative stress was a mechanism for the destruction of the bacteria. They showed and measured hydrogen peroxide released in MgO suspensions and linked the release to the incapacitation of the cells. They identified several mechanisms underlying the action of MgO NPs on bacteria: (1) MgO NPs generate a consistent level of hydrogen peroxide (H_2_O_2_) when in suspension, causing oxidative stress in cells; (2) physical contact between the NPs and the bacterial surface disrupts the bacterial membrane, resulting in membrane leakage; and (3) elevated concentrations of NPs cause substantial membrane damage, release of cellular contents, and irreversible oxidation of biomolecules such as DNA, proteins, and lipids, ultimately leading to cell death.

Aqua Resource Corp. (Fort Walton Beach, Florida, USA) has developed Mg(OH)_2_ nano platelets which they claim have strong activity against bacteria, fungi, and viruses. They have also developed NPs of copper-infused magnesium hydroxide (Cu-infused Mg(OH)_2_) which is even more potent, and they have shown that these NPs killed over 90% of the COVID-19 virus in 4 h. The information on Aqua’s biocidal Mg(OH)_2_ and Cu-infused Mg(OH)_2_ NPs is not available in the open literature, but some information is disclosed here with their permission.

**Figure 11:**
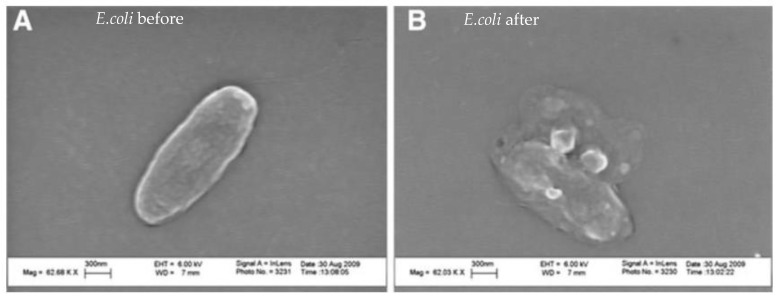
SEM images of *E. coli* cells before (**A**) and after (**B**) exposure to a 2 mg/mL solution of Mg(OH)_2_ nanoparticles for 5 h. Reprinted with permission from ref. [207], Springer Nature License No. 5722931223122. Copyright (2023) Springer Nature.

Aqua reports that Mg(OH)_2_ NPs do not work via leaching ions or penetrating the cell. They kill cells when the platelets come in contact with the cell (Figure 11). These results are consistent with those of other groups (see Figure 8 and Figure 10).

**Figure 12:**
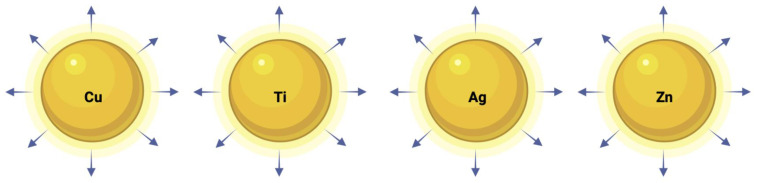
Particles with ionic charge diffusion that kills the microbes.

For oxides of Cu, Ti, Zn, and Ag, metal ions are generated, which diffuse to the microbes and interfere with them, killing them through some of the mechanisms mentioned earlier. Figure 12 shows an illustration of the ionic leaching mechanisms for some types of NPs. The arrows represent their ability to leach out.

**Figure 13:**
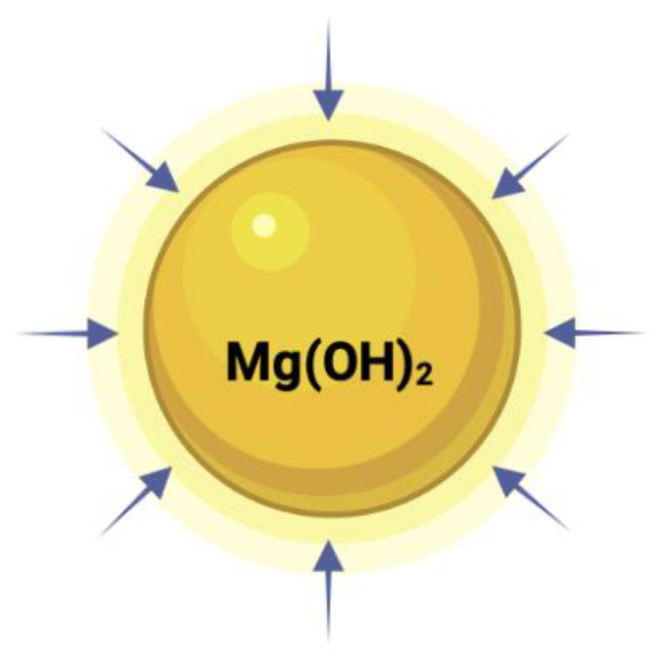
Shows the killing mechanisms of Mg(OH)_2_ which requires direct contact with the microbes.

For Mg(OH)_2_, according to Aqua [207], the dominant killing mechanism is through the contact between the microbes and the crystals (Figure 13); there is no outward diffusion of ions. Some semiconductor NPs generate ROS, and this damages the bacterial cells. The arrows represent their ability to terminate the microbes when they come in contact with the particles.

**Figure 14:**
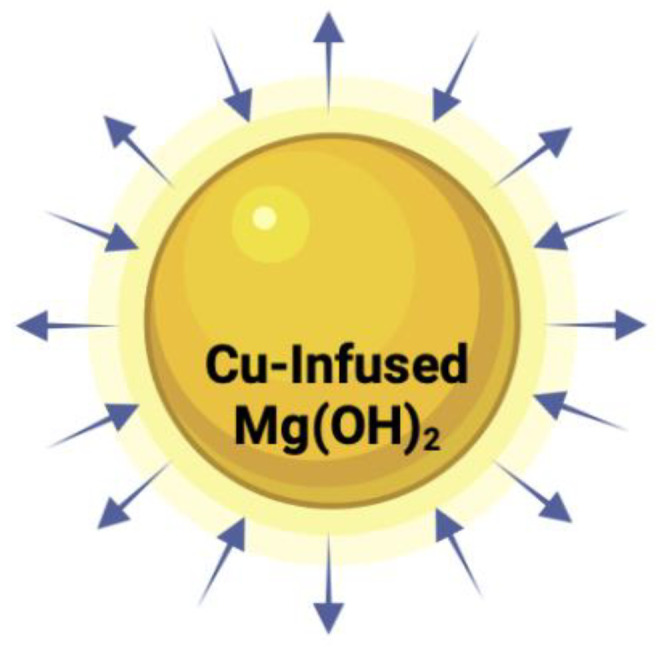
Shows the anticipated killing mechanisms of Cu-infused Mg(OH)_2_ which kill microbes via at least two different modes of mechanism.

In the case of the copper-infused Mg(OH)_2_, Aqua anticipated its extra potency because both methods could operate together: leaching of Cu ions and ROS from Mg(OH)_2_ (Figure 14). The arrows represent their ability to terminate microbes by at least two mechanisms (leaching and non-leaching through direct contact).

**Figure 15:**
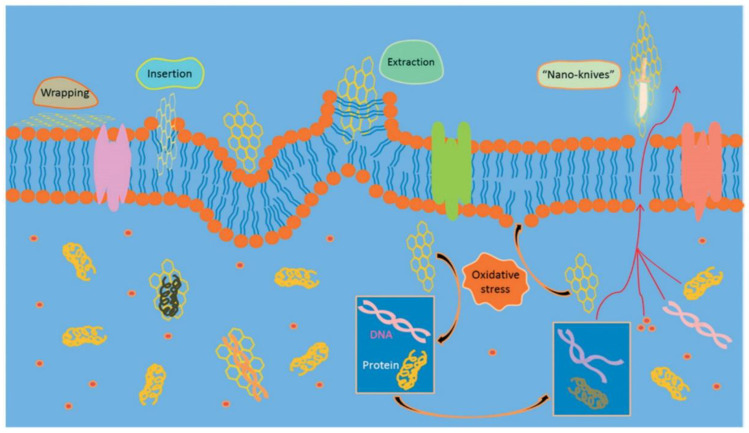
Mechanisms of the antimicrobial activities of graphene materials (GMs). Reprinted with permission from ref. [214], Copyright (2016) American Chemical Society.

Besides the inorganic metal oxides and metals, organic nanomaterials like carbon nanotubes (CNTs) [184], graphene (Gr) [208], graphene oxide (GO) [209], and reduced graphene oxide (RGO) [210] kill microbes [211]. Three mechanisms (Figure 15), wrapping and choking of the microbe via the ultra-thin graphene layers, insertion due to a “Nano-knife effect”, and oxidative stress due to ROS, have been proposed by different authors to explain the antimicrobial actions observed with graphene materials [96]. Zou et al. [212]. reviewed the mechanisms of the antimicrobial activities of graphene and pointed out that while the effect is undeniable, the mechanisms proposed are controversial because of inconsistent experimental designs.

Different authors have proposed identical mechanisms to explain opposite experimental results, and multiple theories of mechanisms have been suggested to elucidate a particular phenomenon. Wang et al. [25]. showed theoretically that a three-layer graphene sheet would be less effective than a monolayer sheet of the same lateral size to pierce through the lipid bilayer of the microbe (nano knife). On the other hand, Mangadlao et al. [213] showed that increasing the number of layers in GO films resulted in stronger antimicrobial activity against *E. coli*. According to their findings, antimicrobial activity is also caused by surface characteristics influenced by the quantity of layers. Thus, from these different works, the picture that emerges is that both the surface and the sharp nano edges of graphene influence its antimicrobial activity.

## 8. Broad-Spectrum or Narrow-Spectrum Microbials and the Shape of Micro-Organisms

Zou et al. note that besides the intrinsic antimicrobial properties of graphene materials, the experimental surroundings (i.e., liquid or solid state, aerobic or anoxic conditions, on a plastic or a metal, and in vitro or in vivo environment) and selected micro-organism genera (i.e., Gram-positive or Gram-negative, round, or rod-shaped) should be considered in assessing the antimicrobial potency.

### 8.1. Bacteria

Single-cell, microscopic organisms usually measure between 1 and 2 µm [215]. Their types depend on shape: spirilla (spiral), cocci (spherical), bacilli (rod), vibrio (comma-shaped), and spirochetes (corkscrew) [216]. The severity of bacterial infections ranges from nonharmful (many live in healthy human bodies, for example, *lactobacillus* and *bifidobacterial*) to potentially fatal (such as *H. pylori* and *S. pneumonia*) [217].

NPs may not be as useful as chemical bleach which destroys all microbes, but, when specific organisms are the problem, a nanocomposite solution has value. The same considerations also apply when assessing other antimicrobial nanomaterials like metal oxide NPs and metal NPs.

According to cell shape, bacteria are classified as coccus (spherical), bacillus (rodlike), spiral, or filamentous. The antimicrobial potency of a substance may depend on cell shape as well. An antimicrobial that kills many types of microorganisms would be the most desirable. The inhibition of various bacterial elements (such as the cell wall, cytoplasmic membrane, and chromosome) is greatly influenced by the shape or structure of specific microorganisms. Among most pathogenic microbes, while the cell wall/membrane, cytoplasm, and nucleus share similarities, their components and configurations remain distinctive. For instance, Gram-negative bacteria like *E. coli* and *P. aeruginosa* possess a thin peptidoglycan layer (approximately 2–3 nm) situated between their inner and outer cell membranes. In contrast, Gram-positive bacteria such as *S. aureus* and *Staphylococcus epidermidis* (*S. epidermidis*) exhibit a thicker peptidoglycan layer (ranging from 20 to 80 nm) within their cell wall, and not all antimicrobials are equally effective with such bacteria.

### 8.2. Fungi

Fungi are another type of microbe that affect polymers. They are a type of eukaryotic microorganism between 4 and 100 μm that acquire nutrients from organic matter [218]. Types include yeast, molds, and smuts [219]. Even though most fungi are harmless, there are some dangerous fungi, such as *Aspergillus* and *Cryptococcus*, which can affect breathing in humans and animals and cause severe sickness. The size and shape of fungi differ depending on the type. For example, yeasts are spherical and have a diameter of 3–5 µm [220].

### 8.3. Viruses

Whereas bacteria are living microorganisms, viruses are technically inert chemical nanoparticles (i.e., non-living) that acquire replication capability when inserted inside a living organism. These are microscopic pieces of genetic information enclosed in a capsid that assumes a helical or icosahedral shape and are mostly between 20 and 100 nm, although some can reach 1000 nm in length [221]. They are usually more dangerous than bacteria. Well known virus types include influenzas, retroviruses, herpesviruses, and coronaviruses [216]. Some viruses, such as *Ebola* and *Zika*, can be fatal to humans. Antibiotics are not effective for viral infections.

## 9. Antimicrobial Testing Protocols

The test methods to measure the survival of micro-organisms are set up under specific physio–chemical conditions, and their relevance or nonrelevance to actual practical conditions needs to be considered. The antimicrobial testing protocols for testing antimicrobial properties of polymers include several procedures, which depend on the type of microorganism being tested.

Antibacterial protocol for nonporous surface (plastic and gloves)

**Figure 16:**
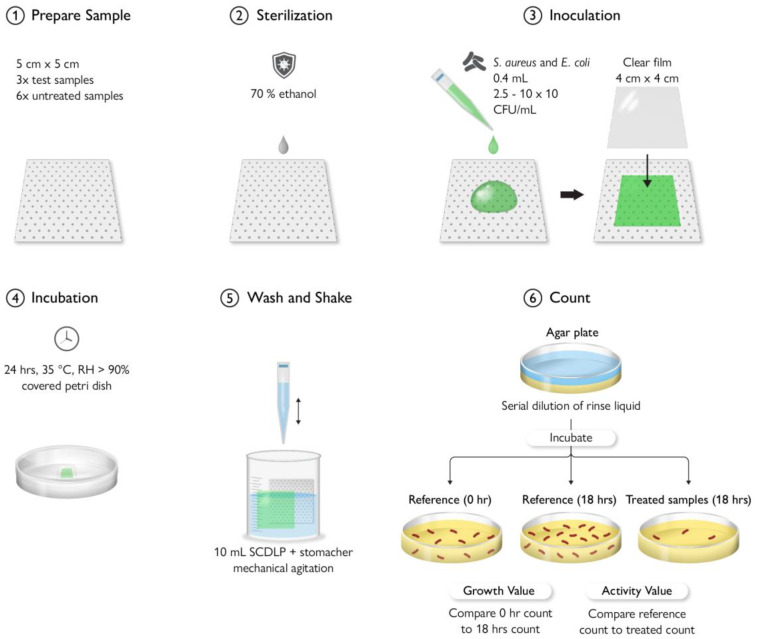
An illustration of the ISO 22196 (measurement of antibacterial activity on plastics surfaces) testing procedure.

ISO standards established by the International Organization for Standardization provide guidelines for the assessment of antibacterial activity on nonporous surfaces such as plastics. One method is direct inoculation, as explained in ISO 22196:2011 [222]. Parameters that must be observed include determining test microorganisms, growing them in a liquid culture medium, and allowing contact between bacteria and products. Additional steps include inoculation through swab, immersion, or touch transfer (swab), removing the bacteria, incubating the material to facilitate bacterial growth, sampling, and colony counting, as well as data reporting and statistical analysis [223]. A detailed illustration for the ISO 22196 (measurement of antibacterial activity on plastics surfaces) is reported in Figure 16.

**Figure 17:**
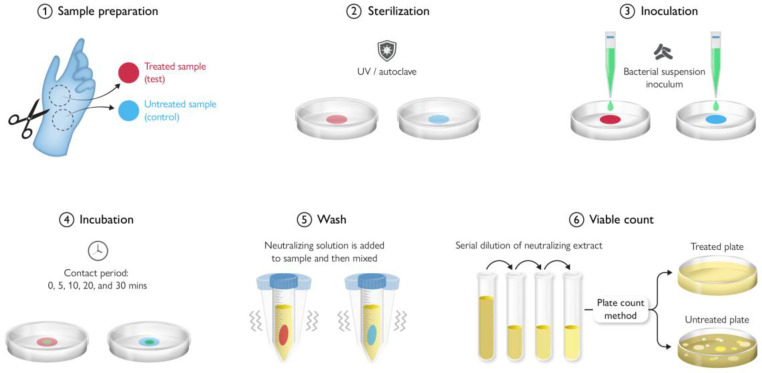
An illustration of the ASTM D7907-14 (determination of bactericidal efficacy on the surface of a medical examination glove) testing procedure.

ASTM D7907-14 [224] is a standardized testing procedure utilized to assess the antibacterial effectiveness of gloves employed in medical examinations. Testing samples are collected from the treated and untreated (control) gloves. Collected samples must either be sterilized with UV or autoclaved before applying the bacterial suspension inoculum. The recommended contact period during the incubation is (0, 5, 10, 20, and 30 min). The neutralizing solution is added to the sample and then mixed for a proper washing. Serial dilutions of neutralizing extract should be applied prior to the plate viable count. A detailed illustration for the ASTM D7907-14 (measurement of antibacterial activity on plastics surfaces) is reported in Figure 17.

Antibacterial protocol for porous surface (textile)

**Figure 18:**
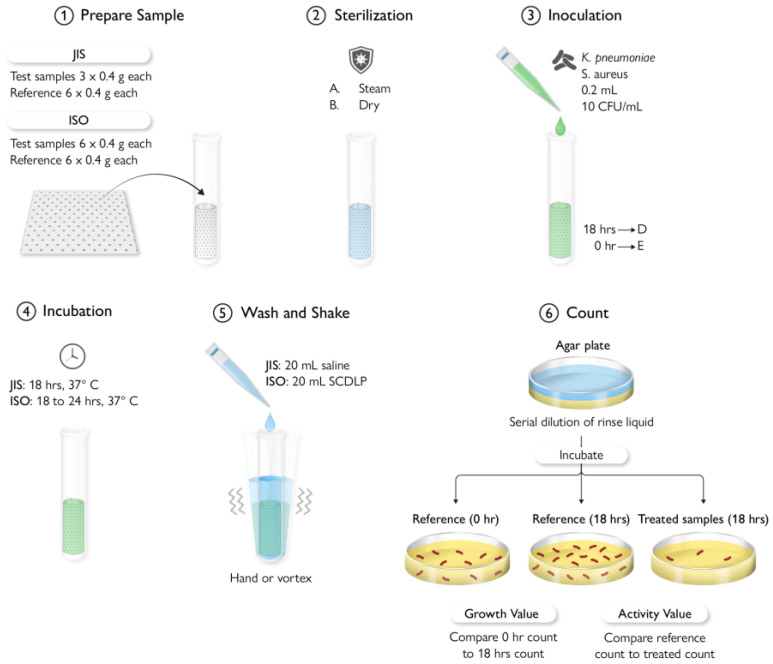
An illustration of the ISO 20743 and JIS L 1902 (determination of antimicrobial activity of textile clothes) testing procedure.

ISO 20743 [225] and JIS L 1902 [226] represent the prevailing evaluation techniques employed for gauging the antimicrobial effectiveness of textile garments. Despite being technically similar, these methods bear distinct nomenclatures due to their association with different organizations. JIS corresponds to the Japanese Industrial Standard (JIS). Both of these testing procedures are conducted to assess the potency of antibacterial-treated clothing. A detailed illustration for the ISO 20743 and JIS L 1902 (determination of antimicrobial activity of textile clothes) is reported in Figure 18.

Another method is the agar diffusion method, explored in ISO/TS 16782: 2016 [227]. It involves transferring antimicrobial agents via diffusion from a chromatogram to an agar plate. After diffusing for a few minutes, the chromatogram is removed, and the plate is incubated [228].

Antiviral protocol for nonporous surface (plastic)

**Figure 19:**
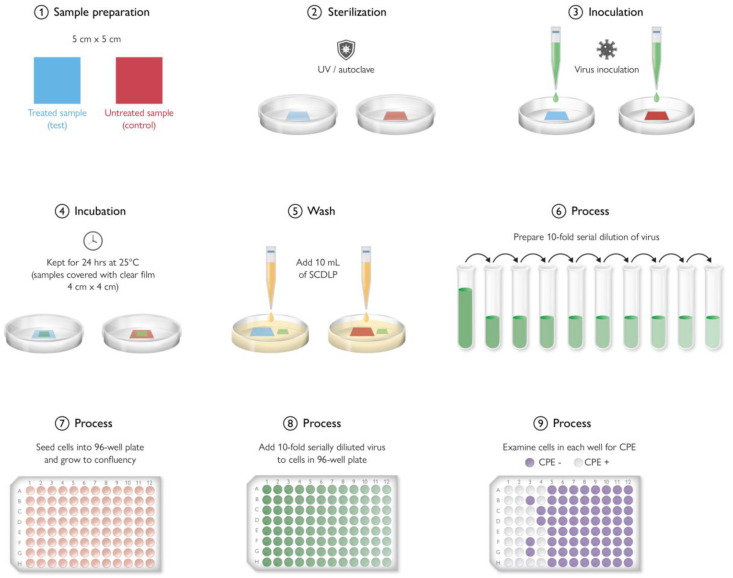
An illustration of the ISO 21702 protocol (proper method for measuring antiviral activity on plastics and other nonporous surfaces of antiviral-treated products) testing procedure.

ISO 21702 [229] is a protocol designed to test the antiviral properties of plastics and nonporous materials. It comprises loading a predetermined virus concentration on treated and reference surfaces [230]. The next step is to leave the surfaces in a humidified chamber at room temperature for about 24 h. Surviving viruses are obtained and washed with liquid media [230]. The recovered amount of infectious virus is then measured, and its infectivity assessed, allowing a researcher to compare the antiviral properties of the treated surface to the reference surface [230]. A detailed illustration for the ISO 21702 (the proper method for measuring antiviral activity on plastics and other nonporous surfaces of antiviral-treated products) is reported in Figure 19.

Antiviral protocol for porous surface (textile)

**Figure 20:**
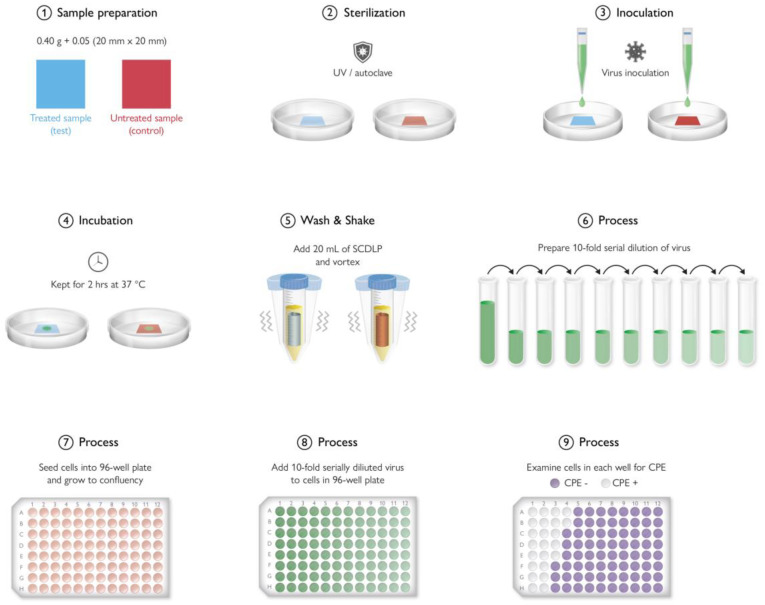
An illustration of the ISO 18184 (testing method for the determination of the antiviral activity of textile products) testing procedure.

ISO 18184 [231] serves as a specific test for antiviral properties in textiles and porous materials, gauging their virucidal antimicrobial efficacy. This standard is tailored to evaluate the capability of these materials to eliminate viruses. It involves exposing the materials to viruses for durations spanning from 2 h up to just under 24 h as per the specified exposure times. A detailed illustration for the ISO 18184 (testing method for the determination of the antiviral activity of textile products) is reported in Figure 20.

Antifungal protocol for nonporous surface (plastic)

The ASTM G21 [232] test procedure is utilized for assessing the ability of synthetic polymer and plastic materials to withstand fungal proliferation. Typically, pure synthetic polymer materials inherently resist fungal growth as they lack properties that serve as a carbon source to support the development of fungi. Nevertheless, various supplementary elements or additives such as plasticizers, lubricants, stabilizers, cellulose, and colorants incorporated in synthetic plastic materials can instigate fungal growth and contribute to their decay. Hence, conducting antifungal resistance tests becomes crucial to guarantee the longevity and endurance of plastic materials that might be susceptible to fungal growth, ensuring their overall durability.

Antifungal protocol for porous surface (textile)

The AATCC 30 [233] standard method is employed to assess the effectiveness of textile materials treated with fungicidal substances. These fungicidal finishes are applied to textiles to confer protection against mildew and decay. The AATCC 30 testing procedure is applicable to various textile materials, encompassing cotton, rayon, nylon, silk, wool, linen, polyester, co-acrylic, spandex, and viscose, as well as items like sandbags, tarpaulins, tents, and more.

**Figure 21:**
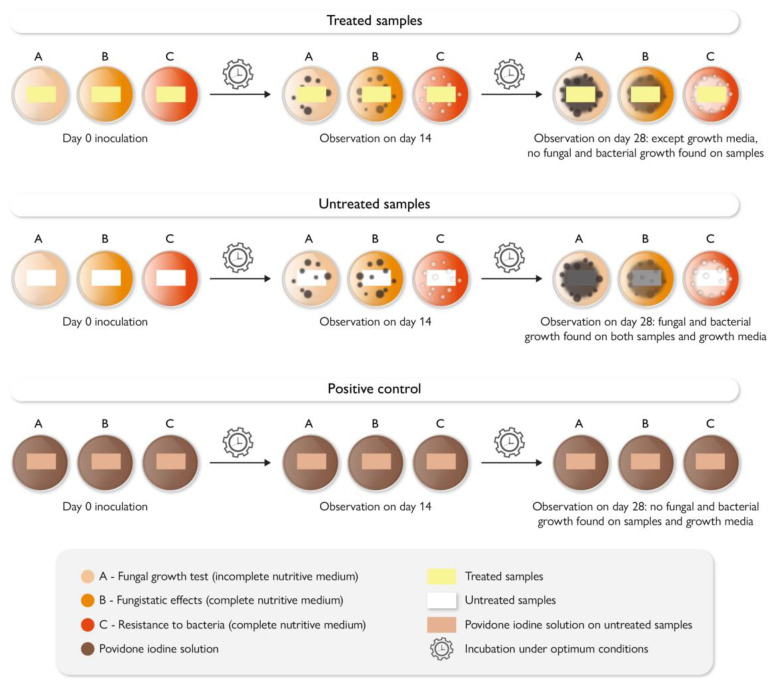
An illustration of the ISO 846 (evaluation of the action of micro-organisms on plastics) testing procedure.

ISO 846 [234] is utilized to evaluate the degradation of plastics caused by the presence of fungi, bacteria, and microorganisms found in soil. The colonization of microorganisms on plastic surfaces is typically induced by external elements such as temperature, humidity, and gaseous surroundings. The degradation of plastic materials by bacteria and fungi occurs through two primary mechanisms: (1) Direct action–plastics serve as a substrate for the growth of microorganisms, leading to the deterioration of the plastic itself. (2) Indirect action–metabolic byproducts produced by microorganisms contribute to the degradation of plastic. These processes ultimately impact the physical characteristics and functionality of products, resulting in a reduced shelf life. The ISO 846 test method is specifically designed to assess the degradation of impermeable plastic materials against a set of test microorganisms. Evaluation involves parameters such as visual inspection, alterations in mass, or changes in other physical attributes. However, it is important to note that this test is not intended to measure the biodegradability of plastic materials. A detailed illustration for ISO 846 (evaluation of the action of microorganisms on plastics) is reported in Figure 21.

**Figure 22:**
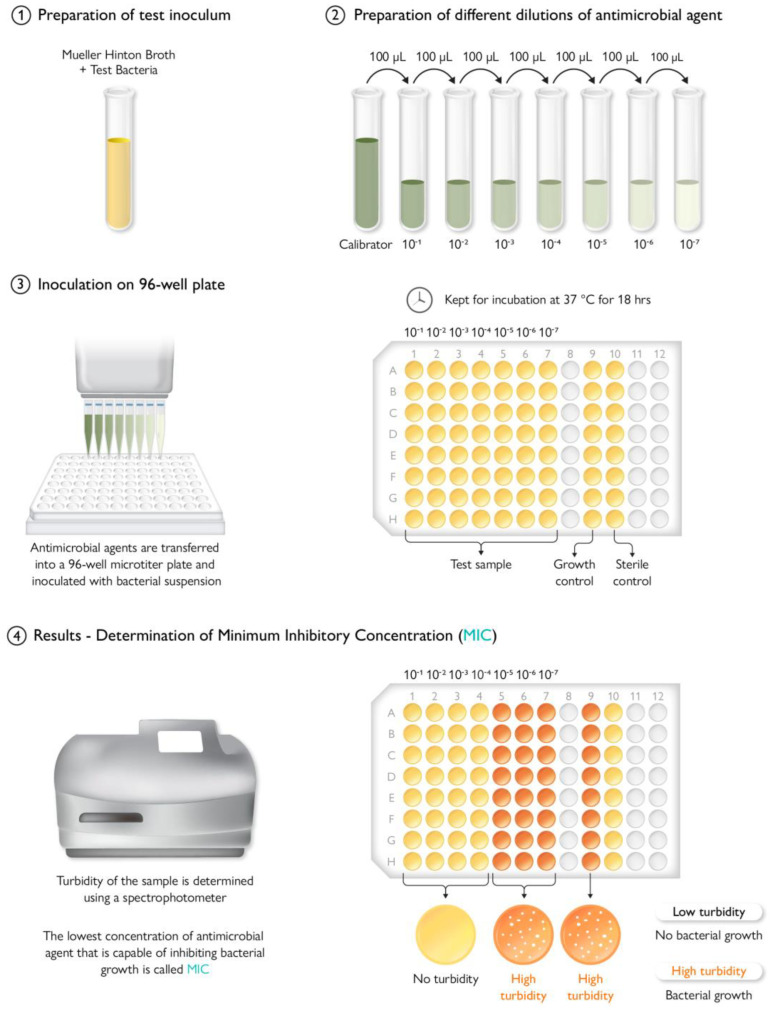
An illustration of the minimum inhibitory concentration (MIC) test procedure.

ISO 16256:2021 [235] provides an international standard for testing the activity of antimicrobial agents against disease-causing yeast fungi. The first step is the preparation of yeast cultures, broth medium, and antimicrobial agents. Then, one inoculates the yeast into the medium. The cultures should be incubated for a particular timeframe. The step suggests determining the agent’s minimum inhibitory concentration (MIC). The last step is analyzing MIC results, which can be performed using EUCAST or CLSI. EUCAST involves spectrophotometrically calculating yeast growth after incubation, while CLSI involves visual assessment of the growth [236]. A detailed illustration for the minimum inhibitory concentration (MIC) is reported in Figure 22.

**Figure 23:**
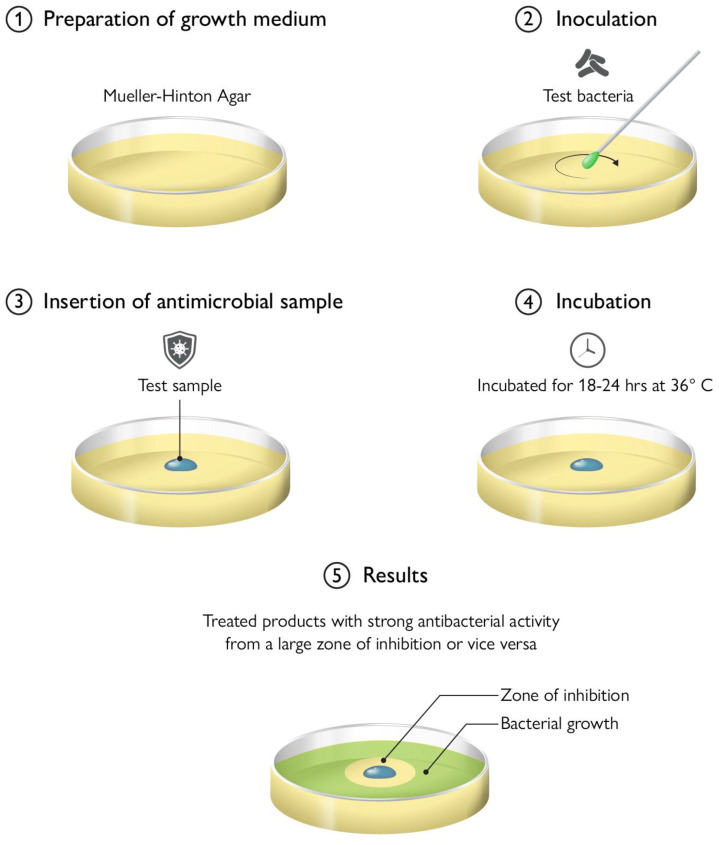
An illustration of the Zone of Inhibition test procedure.

The Zone of Inhibition test, also known as the Kirby–Bauer test, is employed to assess the susceptibility or resistance of pathogenic bacteria to antibacterial agents. Compared to alternative laboratory testing approaches, the Zone of Inhibition test is a cost-effective and rapid method for evaluating antibacterial activity. A significant benefit of this test is its versatility, as it can be applied to various samples of antibacterial products, including treated textiles, polymers, disinfectants, and antibiotics. A detailed illustration for the Zone of Inhibition (ZOI) test is reported in Figure 23.

Difference Between Minimum Inhibitory Concentration (MIC) and Zone of Inhibition (ZOI)

Minimum inhibitory concentration (MIC) refers to the lowest concentration of an antimicrobial substance necessary to prevent visible microorganism growth following incubation. This test assesses the effectiveness of a test agent against a particular bacterium. The Zone of Inhibition (ZOI) is a clear circular region surrounding antimicrobial discs where bacteria cannot grow. Both MIC and ZOI are inversely related and hinge on the susceptibility of the test organism to the antimicrobial substance. Greater susceptibility leads to a larger ZOI and a lower MIC. Conversely, a higher MIC value coupled with a smaller ZOI suggests bacterial resistance to antimicrobial agents. In assessing the antimicrobial efficacy of treated products, quantitative tests prove advantageous as they yield more precise and distinct outcomes.

**Figure 24:**
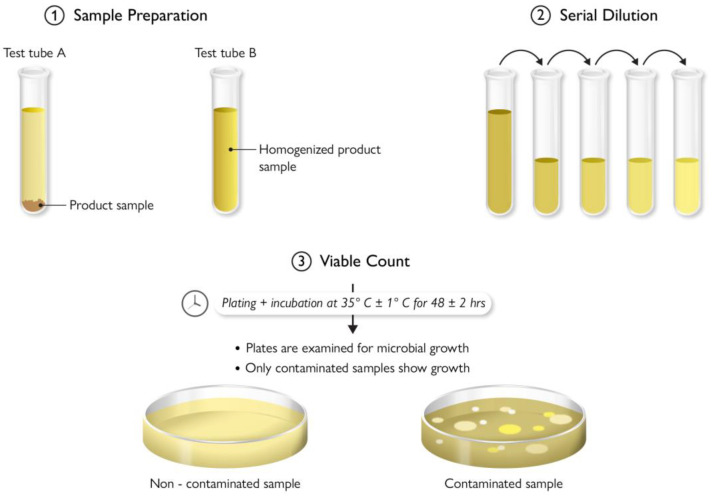
An illustration of the aerobic plate count (APC) test procedure (antibacterial).

Aerobic plate count (APC) is a method used to approximate the quantity of viable microorganisms present in specific test samples or products. This test determines the population of aerobic mesophilic bacteria. Aerobic plate count is alternatively referred to as the standard plate count, aerobic mesophilic count, total plate count, or aerobic colony count. However, a limitation of this method is its inability to differentiate between various bacterial colonies within a given sample. A detailed illustration for the aerobic plate count (APC) is reported in Figure 24.

**Figure 25:**
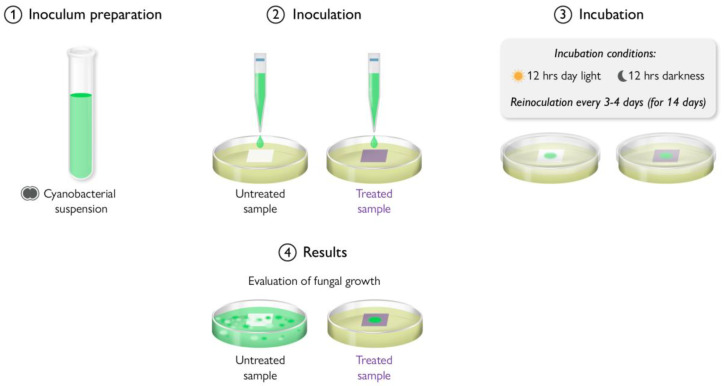
An illustration of the ASTM G29 (determination of algal resistance of plastic films) testing procedure.

The ASTM G29 [237] test method is employed to assess the effectiveness of surfaces treated with antimicrobial agents in preventing the growth of algae. Algae typically thrive in damp or moist environments and are commonly found in or around water bodies. Human-made structures that retain water, such as swimming pools, irrigation ditches, water storage tanks, and artificial ponds, often utilize plastic films. Over time, algae can proliferate, forming substantial microfilms that can penetrate these structures and lead to unappealing deposits. To mitigate the spread of algae in artificial water environments, coatings or films with antifungal properties have been developed to resist algae growth. The objective of the ASTM G29 test is to evaluate the level of surface protection against algae by utilizing plastic films integrated with antimicrobial properties. A detailed illustration for the ASTM G29 (determination of algal resistance of plastic films) testing procedure is reported in Figure 25.

### 9.1. In Vivo Testing Method

In vivo testing involves performing tests, procedures, and experiments on or in living organisms [238]. Concerning microbial polymers, the testing method is used to evaluate the effectiveness of the protection offered by antimicrobial polymers. Features include complex interactions and practical settings [238]. The advantages are specificity and detail as well as mirroring real-life situations, while disadvantages include higher costs, increased complexity, and longer duration [238].

### 9.2. In Vitro Testing Method

In vitro testing involves performing experiments in controlled lab settings, usually involving isolated microbes, to assess the protection offered by antimicrobial polymers or coatings [238]. The laboratory setting allows researchers to explore how antimicrobial materials interact with test organisms [238]. Features of in vitro testing include isolation and simple interactions [238]. Advantages include cost-effectiveness, speed, reliability, and simplicity [239]. Disadvantages include the inability to capture the innate complexity of organ systems, limited evaluation of impacts, and challenges in simulating the impacts of long-term exposures [240].

The real understanding of the test method used enables control of the growth of micro-organisms in real situations such as a plastic surface (packaging film, medical tool, etc.). Whether the nanocomposite will work in an actual device requires correlating the test with the design of the article. In order to be considered for practical use in healthcare or food packaging, plastic nanocomposites should not be harmful to human cells.

A survey of the literature shows there is likely to be uncertainty about the mechanism (especially when there is more than one). The knowledge across disciplines that is needed to interpret the results is a factor in the field of antimicrobial nanocomposites, which can lead to contradictory explanations for the mechanism.

### 9.3. Antimicrobial Plastic Results Variation

There is notable variation in antimicrobial results for polymeric and microbial surfaces, and they can be attributed to reasons such as testing methodologies, types of antimicrobial agents, environmental settings, solubility, hydrophilic properties, and exposure time [241,242,243]. For example, results of how much antimicrobial protection is offered by antimicrobial coating on a polymer such as chitosan may differ due to the environment to which it is exposed [243]. Chitosan from a dumpster is likely to contain a different microbe concentration than from another dumpster, leading to varying degrees of antimicrobial protection offered by coating [243]. Hydrophilic properties can also contribute to the variation. Hydrophilic surfaces attract water, while hydrophobic ones repel water. Since water is vital for microbe growth and development, hydrophilic surfaces are more likely to contain more microbial growth [244]. The type of agent is also relevant. Leachable agents release antimicrobial compounds from the polymer matrix, while nonleachable ones do not since they are embedded within the polymer, where no direct interaction would happen between the NPs and the microbes. They offer varying degrees of protection over time. As a result, testing for microbial activity may have vastly different outcomes [245]. The solubility of an agent also contributes to the variation in results. Soluble agents may be more effective when applied to a surface since they kill all existing microbes they interact with [246]. However, they dry away shortly afterward, lessening antimicrobial protection. Nonsoluble agents kill existing microbes and those that come into contact with the surface later. Therefore, the results may differ. Exposure time also matters. The longer the exposure time, the higher the likelihood of microbes being killed even when using lower concentrations [247]. Thus, the testing method has to be thoroughly matched to the antimicrobial/plastic system being investigated and care must be taken to ensure the test method does not create the conditions for a desirable antimicrobial result.

## 10. Toxicity

The use of NPs in polymers or as coating is critical for protecting human safety and the environment. Given the health risks of NPs, popular methods of testing their impact range from lactate dehydrogenase and endotoxin signaling to ROS detection and apoptosis, with no specific approach in the numerous protocols [248]. As a complementary test, experts conduct a spectroscopic exam to assess the permeation of the epithelial barrier and determine the extent to which NPs deposit themselves in living cells [248]. The procedure comprises preparation, selecting the test surface, exposure, evaluating the impact of the particles on the surface, assessing the effect on particles, and data analysis.

## 11. Antimicrobial Market Size

The antimicrobial polymer market has experienced significant growth in recent years. According to Fortune Business Insights (2023), the worldwide antimicrobial polymer market size was USD 41.2 billion in 2022, USD 43.9 billion in 2023, and estimated to reach USD 73.7 billion by 2030 [249]. The increasing demand for antimicrobial polymers can be attributed to numerous advantages compared to untreated plastics, which are attacked by algae, fungi, and bacteria. The increasing demand can also be attributed to better performance, durability, and diverse applications. The use of polymers will increase as their role in automotive manufacturing and packaging grows. The recent COVID pandemic, which was of global significance, has also spurred interest in methods to prevent the spread of microorganisms.

## 12. Conclusions

This review has covered various uses of polymers for antimicrobial control. In one aspect, polymers are hosts for storing biocides/antibiotics for controlled and targeted drug release. This method is useful when the person is already infected. Another type is in the fabrication of “polymeric biocides” which have moieties in the polymer chain capable of killing microbes. Further, the adhesion of microbes to object surfaces can be prevented by engineering the surfaces which prevents the growth of microbes in areas handled by people. Biocidal polymers are nanocomposites capable of microbe destruction through multiple methods. They could offer a viable alternative to antibiotics and disinfectants. With biocides that show multiple mechanisms of killing microbes such as Ag^+^, the pathogens cannot develop resistance as with antibiotics.

Medical devices, implants, wound dressings, and contact lenses require microbe-free surfaces, and coating them with polymers possessing antiadhesive and/or antimicrobial properties is one of the approaches to rendering the required microbe-free properties. Design choices include the synthesis of polymers with only antiadhesive (biopassive) or antimicrobial (bioactive) properties. Further, combining both methods is possible. However, in most of these approaches, the polymers are not standard commodity plastics used for fabricating engineered articles. The biocide-releasing and biocidal polymers (like PEG, PEI, etc.) described in the literature can often be applied only as coatings to both polymer and nonpolymer surfaces.

## Figures and Tables

**Table 1:** Studies involving polymer-based nanocomposites that demonstrate effective use against a range of microorganisms on different surfaces. In most studies, the polymer matrix chosen is not a common, inert thermoplastic but polymers that have biocidal properties, such as PEG, PEI, etc. Reproduced from [176], open access. Copyright (2021) Iulia Babutan, Alexandra-Delia Lucaci, and Ioan Botiz.

Blend/Composite	Configuration/ Nanostructure	Dimension	Antimicrobial Mechanism	Efficacy	Microbe of Interest	Ref.
PMMA/Ag, PTBAM/Ag	Nanofibers	40 nm diameter, 10 µm long	Bioactive reinforced	-	*E. coli*, *S. aureus*	[177]
PEI/Ag	NPs grafted on SAM	10–14 nm thick (total)	Bioactive reinforced	~6 log 0.86 log	*E. coli* *S. aureus*	[178]
PVDF-g-PCBMA/Ag	Pores/brushes	-	Bioactive reinforced	-	*E. coli*, *S. aureus*	[179]
PLA/PEG, PLA/PEG/Ag	Films; NPs	~40 µm thick 25 nm thick	Biopassive + bioactive	-	*E. coli*, *S. aureus*	[180]
P2VP-b-PEG	Smart micelles	60 nm (unloaded)	Bioactive		-	[181]
PEI/Cu	Positively charged NPs	34 nm radius	Bioactive reinforced	87% 96% 80%	*E. coli* *P. aeruginosa* *S. aureus*	[182]
Pectin-PEI-Cu	Films with Cu NPs	100 µm thick	Bioactive reinforced	-	*S. aureus*, *E. coli*	[183]
PDMEMA-MWCNTs	Nanotubes	26 nm diameter	Bioactive reinforced	42% -	*E. coli* *S. aureus* *E. coli*	[184]
MWCNTs-APPI/MWCNTs-APPI-Ag Nps	Nanotubes Ag NPs	15 nm diameter	Bioactive reinforced	96%/99% 96/99% 87%/83%	*B. subtilis* *S. aureus* *E. coli*	[185]
PE/PEG/GO-NH_2_	Films	-	Bioactive reinforced	90%	*E. coli*	[186]

An example is to fabricate antibacterial coatings for implantable devices [25]. Biocidal metals like Nano Ag (1–100 nm diameter) overcome the ability of bacteria to develop resistance because they operate with multiple and simultaneous antibacterial mechanisms. These mechanisms reduce the chance of a bacterium developing mutations on multiple genes [25].

**Table 2:** List of various radical forms of reactive oxygen species (ROS) formed by NPs.

The Radicals (Not Stable)	The Nonradicals (Stable)
Superoxide (O_2_∙^−^)	Hydrogen peroxide (H_2_O_2_)
Hydroxyl (OH∙)	Hypochlorous acid
Peroxyl, alkoxyl (RO_2_∙,RO∙)	Ozone (O_3_)
Oxides of nitrogen (NO∙,NO_2_∙)	Singlet oxygen (1O_2_)

## Data Availability

Not applicable.
